# Host and Species-Specificities of Pattern Recognition Receptors Upon Infection With *Leptospira interrogans*

**DOI:** 10.3389/fcimb.2022.932137

**Published:** 2022-07-22

**Authors:** Delphine Bonhomme, Catherine Werts

**Affiliations:** Institut Pasteur, Université de Paris, CNRS UMR2001, INSERM U1306, Unité de Biologie et Génétique de la Paroi Bactérienne, Paris, France

**Keywords:** *Leptospira*, *zoonosis*, MAMPs, TLRs, NOD, NLRP3, host-specificity, leptospirosis

## Abstract

Leptospirosis is a zoonotic infectious disease affecting all vertebrates. It is caused by species of the genus *Leptospira*, among which are the highly pathogenic *L. interrogans*. Different mammals can be either resistant or susceptible to the disease which can present a large variety of symptoms. Humans are mostly asymptomatic after infection but can have in some cases symptoms varying from a flu-like syndrome to more severe forms such as Weil’s disease, potentially leading to multiorgan failure and death. Similarly, cattle, pigs, and horses can suffer from acute forms of the disease, including morbidity, abortion, and uveitis. On the other hand, mice and rats are resistant to leptospirosis despite chronical colonization of the kidneys, excreting leptospires in urine and contributing to the transmission of the bacteria. To this date, the immune mechanisms that determine the severity of the infection and that confer susceptibility to leptospirosis remain enigmatic. To our interest, differential immune sensing of leptospires through the activation of or escape from pattern recognition receptors (PRRs) by microbe-associated molecular patterns (MAMPs) has recently been described. In this review, we will summarize these findings that suggest that in various hosts, leptospires differentially escape recognition by some Toll-like and NOD-like receptors, including TLR4, TLR5, and NOD1, although TLR2 and NLRP3 responses are conserved independently of the host. Overall, we hypothesize that these innate immune mechanisms could play a role in determining host susceptibility to leptospirosis and suggest a central, yet complex, role for TLR4.

## Introduction

Leptospirosis is a neglected zoonotic disease causing around 1 million human cases and 60,000 deaths *per* year worldwide (Costa et al., 2015). Outbreaks are linked to environmental events, such as floods (Ritter et al., 2018), and are most likely to increase with climate change. It is currently re- and newly emerging, and the major challenges in the field are the lack of a cross-protective vaccine between serovars and the poor diagnosis tools available.

### Leptospirosis—Zoonotic Cycle

**Figure 1:**
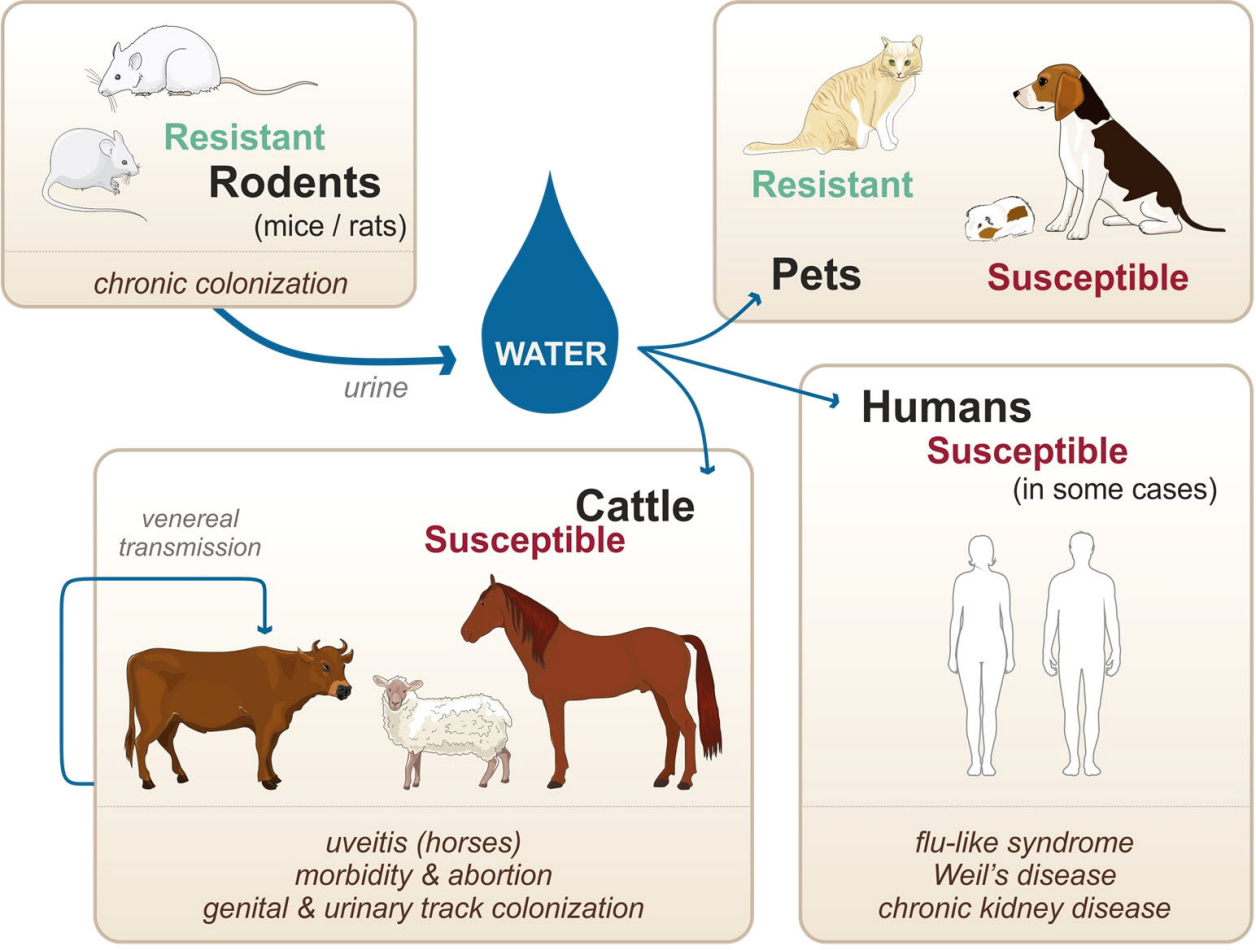
Zoonotic cycle of leptospirosis, susceptibility of various accidental hosts, and transmission modes. Adapted from Adler, 2015.

Unlike other spirochetes, such as *Borrelia burgdorferi* and *Treponema pallidum* which are obligate parasitic bacteria, pathogenic *Leptospira* survive in the environment as well as within the infected vertebrate hosts. Natural reservoirs are in most cases asymptomatic rodents (namely, mice and rats considered as resistant hosts) that are chronically colonized in their kidneys upon infection and persistently excrete the bacteria in their urine (**Figure 1**) (Ko et al., 2009). Once excreted in the environment, leptospires can infect susceptible hosts, such as human, cattle, and pets. Infection occurs through abrased or damaged skin and mucosa. In the host, leptospires can cause a systemic infection, leading to severe illness in some cases (**Figure 1**). Furthermore, venereal transmission has been suggested in infected cattle and goat (Lilenbaum et al., 2008). Not all leptospires spp. are pathogenic, and recent genomic studies have reclassified and clustered more than 60 species in groups and clades according to their pathogenicity in human and animal hosts (Vincent et al., 2019). Pathogenic species belonging to the P1 clade and responsible for severe forms of leptospirosis are *L. interrogans*, *L. kirschneri*, *L. noguchii*, *L. santarosai*, *L. mayottensis*, *L. borgpetersenii*, *L. alexanderi*, *L. weilii*, *L. alstonii*, *L. dzianensis*, *L. barantonii*, *L. kmetyi*, *L. tipperaryensis*, *L. putramalaysiae*, *L. adleri*, *L. ellisii*, and *L. gomenensis (* Vincent et al., 2019), although *L. interrogans* is responsible for the more severe diseases.

### Leptospirosis—Various Hosts and Symptoms

All vertebrates can potentially be infected by leptospires, and numerous hosts, such as humans, are susceptible to the disease, with the incubation period of leptospirosis varying greatly from a few days to a few weeks (Adler, 2015). The disease presents itself with an abrupt undifferentiated febrile syndrome, first characterized by fever, chills, muscle pain, and headache (Adler, 2015). In some patients, infection with leptospires can cause more severe forms of the disease, characterized by multiorgan failure (kidneys, liver, lungs, brain) at the late stage of the disease (Adler, 2015). The most distinguishable form of severe leptospirosis is kidney failure associated with jaundice, called Weil’s disease. Overall, the general symptoms of leptospirosis are common to many other infectious diseases, hence complicating the diagnosis.

Among other susceptible hosts, ruminants are particularly affected and infection results in morbidity, abortion, and infertility, with some documented cases of severe illness (Ellis et al., 1985; Ellis et al., 1986; Adler, 2015). Similarly, pigs and horses suffer from various symptoms, with the peculiarity that horses can have recurrent uveitis, potentially caused by leptospiral biofilms (Ellis et al., 1983; Adler, 2015; Ackermann et al., 2021). In the case of pets, dogs are susceptible, and infection can result in an acute icteric form with severe pulmonary (Moore et al., 2006; Adler, 2015). However, dogs can also be chronically infected, with clinical signs including chronic gastritis (Adler, 2015). In this context, it should be emphasized that different hosts are preferentially infected with different species (spp.) and serovars of leptospires. For instance, *Leptospira borgpetersenii* serovar Hardjo-Bovis is mainly isolated from cattle whereas *Leptospira interrogans* serovar Canicola is mainly isolated from dogs (Adler, 2015). Additionally, almost all infected animals are chronically colonized upon infection. In the case of cattle, pigs, and horses, leptospires colonize the genital tract as well as the kidneys. Such colonization allows for venereal transmission of the pathogens as well as shedding of the pathogen into the environment *via* the urine (Lilenbaum et al., 2008) (**Figure 1**). In contrast to the mammalian species mentioned above, mice and rats do not show signs of an acute form of leptospirosis and do not present symptoms of severe illness upon natural infection (Adler, 2015). However, upon experimental infection, leptospires can colonize the proximal tubules in their kidneys and remain present throughout the lifetime of the mice (Ratet et al., 2014). Although mice present very few symptoms of the disease, the chronic renal carriage induces mild fibrosis (Fanton d’Andon et al., 2014; Ferrer et al., 2018). Such colonization plays a key role in the zoonotic cycle of leptospirosis, as leptospires get excreted in the urine of the rodents, hence contaminating the environment. Of note, not all rodents are resistant to the disease: guinea pigs, gerbils, and hamsters for instance are used as experimental models of human acute leptospirosis since they are sensitive and can die of acute infection (Lourdault et al., 2009). When they survive the infection, hamsters also present renal fibrosis (Matsui et al., 2016; Gomes et al., 2018). Furthermore, studies suggest that cats are very resistant to acute leptospirosis although they can be colonized (Holzapfel et al., 2021), but very few clinical cases have been described (Adler, 2015).

Leptospirosis has therefore many consequences for both humans and animals and represents a major health and economic burden for agriculture and breeding. In addition to the details on human leptospirosis given above, information concerning animal susceptibility, symptoms, chronicity, and infecting species and serovars is summarized in **Table 1**.

**Table 1:** Susceptibility and symptoms of various animal hosts upon natural infection by Leptospira spp.

Class	Animal	Susceptibility	Chronicity	SYMPTOMS	Infecting species/serovars
Rodent	**Mouse** (Paiva-Cardoso M das et al., 2013; Moinet et al., 2021)	**Resistant**	**+** kidneys	NA	*L. borgpetersenii* Ballum
Rodent	**Rat** (Hathaway and Blackmore, 1981; Heuser et al., 2017; Costa et al., 2021; Moinet et al., 2021)	**Resistant**	**+** kidneys	NA	*L. interrogans* Copenhageni & Icterohaemorrhagiae *L. borgpetersenii* Ballum
Rodent	**Guinea pig** (Rigby, 1976; Monte et al., 2013)	**Susceptible**	*undetermined*	Acute illness Icteric forms	*L. interrogans* Icterohaemorrhagiae
Rodent	**Hamster** (Sebek et al., 1987; Desai et al., 2009)	**Susceptible**	**+** kidneys	Acute illness Icteric forms	*L. interrogans* Pomona
Ruminant	**Cattle** (Ellis et al., 1985; Ellis et al., 1986)	**Susceptible**	**+** genital tractus kidneys	Abortion Premature birth Some severe cases	*L. borgpetersenii* Hardjo-Bovis *L. interrogans* Hardjo
Ruminant	**Deer** (Subharat et al., 2010)	**Susceptible**	*Undetermined*	*Undetermined*	*L. interrogans* Pomona & Hardjo
Ruminant	**Sheep** (Ellis et al., 1983) (Hamond et al., 2019)	**Intermediate**	**+** genital tractus kidneys	Sporadic outbreaks of severe cases/abortion Infertility	*L. interrogans* Pomona, Icterohaemorrhagiae, Australis & Sejroe
Ruminant	**Goat** (Leonvizcaino et al., 1987)	**Susceptible**	*Undetermined*	Abortion Some severe cases	*L. interrogans* Australis, Grippotyphosa, Hebdomadis, Sejroe & Pomona
Swine	**Pig** (Ellis et al., 1986)	**Intermediate**	**+** genital tractus kidneys	Abortion Some severe cases Infertility	*L. interrogans* Pomona, Australis & Tarassovi
Equid	**Horse** (Ellis et al., 1983)	**Sensitive**	**+** genital tractus kidneys	Uveitis, abortion Some severe cases Infertility	*L. interrogans* Pomona, Grippotyphosa, Icterohaemorrhagiae, Autumnalis, Sejroe & Canicola
Pet	**Dog** (Moore et al., 2006)	**Susceptible**	**+** kidneys	Icteric forms Severe pulmonary forms Chronic uveitis/gastritis	*L. interrogans* Canicola, Pomona, Icterohaemorrhagiae, Bratislava & Autumnalis
Pet	**Cat** (Larsson et al., 1985; Holzapfel et al., 2021)	**Resistant**	*Undetermined*	NA	*undetermined*

Adapted from Adler, 2015 and other references indicated.

### *Leptospira interrogans*—Atypical Cell Wall

**Figure 2:**
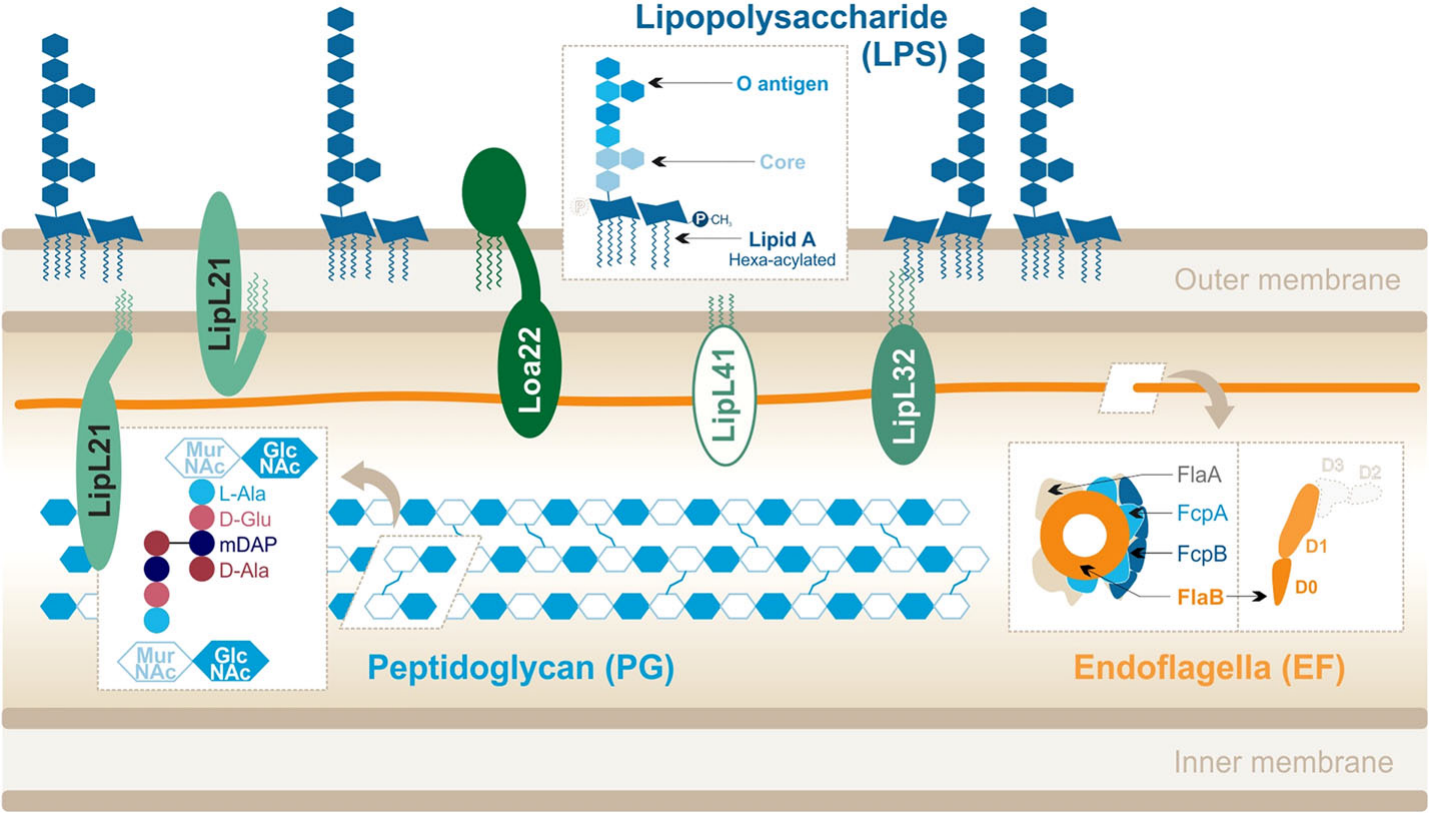
Leptospira interrogans cell wall and MAMPs: peptidoglycan (PG), endoflagella (EF), abundant lipoproteins and atypical lipopolysaccharide (LPS). Adapted from Que-Gewirth et al. (2004), Haake and Zückert (2015), Ratet et al. (2017), Gibson et al. (2020).

*Leptospira* spp. responsible for leptospirosis are diderm bacteria with an atypical cell wall composition (**Figure 2**). Although they possess two membranes, leptospires exhibit an intermediate staining phenotype and can therefore not be classified as Gram-negative bacteria.

Their peptidoglycan (PG) layer is located slightly closer to the inner membrane than in other bacteria (Cameron, 2015). In addition, leptospires are equipped with two endoflagella (EFs), embedded in the periplasm, that confer them great motility. Leptospires are also particularly rich in outer membrane proteins (OMPs), including numerous lipoproteins (Haake and Zückert, 2015) which are the most abundant components of the leptospiral cell wall. Finally, to our interest, *Leptospira* spp. differ from most other spirochetes because they possess lipopolysaccharides (LPSs) anchored in their outer membrane (Vinh et al., 1986; Werts et al., 2001). The structure of the leptospiral LPS has not been extensively studied, unlike LPS from *Escherichia coli* or *Salmonella minnesota*. LPS is an assortment of complex molecules that have a tripartite structure: **(i)** lipid A that is composed of a disaccharide with acyl chains anchoring the LPS in the outer membrane of the bacteria, **(ii)** the core (composed of sugars) which forms the first part of the carbohydrate component, and **(iii)** the O antigen that is an assembly of repeated sugar units protruding outside of the bacteria which forms the second (**Figure 2**).

The structure of the leptospiral lipid A (Que-Gewirth et al., 2004) revealed that disaccharide is composed of 2,3-diamino-2,3-dideoxy-D-glucopyranose units. It is hexa-acylated with two R-3-hydroxylaurates (in 3 and 3′) and two R-3-hydroxypalmitate (in 2 and 2′) with the peculiarity of having two secondary unsaturated acyl chains C12:1 in 2′ and C14:1 in 3′. Furthermore, the four primary acyl chains are amine-linked because of the substitution of carbon atoms by nitrogen atoms in 3 and 3′. Finally, unlike *E. coli* LPS, the leptospiral LPS is lacking a 4′-phosphate group and the 1-phosphate group is methylated (**Figure 2**) and therefore does not present the usual negative charges. The peculiarities of the *L. interrogans* lipid A are conserved in several pathogenic serovars (Lai, Icterohaemorrhagiae, and Manilae) (Que-Gewirth et al., 2004; Eshghi et al., 2015; Novak et al., 2022).

The LPS from *L. interrogans* has very little amount of the traditional KDO moiety (Vinh et al., 1986; Patra et al., 2015), and it is currently hypothesized that *Leptospira* spp. could use other forms of sugars such as KDO (Vinh et al., 1986; Patra et al., 2015), as is the case for *Acinetobacter* and *Burkholderia* spp. (Erridge et al., 2002). Also, very little is known regarding the structure of the O antigen. The O antigen of *E. coli* is composed of repeated sugar units that make a very characteristic ladder-like pattern when revealed on acrylamide gel. In the case of *Leptospira*, arabinose, xylose, mannose, galactose, and mannoheptose were found in several O antigens (Patra et al., 2015); however, the smear-like bands visible in silver-stain SDS-PAGE analyses suggest that the leptospiral O antigen is much more complex than the repeated sugar units present in *E. coli* or *S. enterica* LPS (Cinco et al., 1986; Bonhomme and Werts, 2020). Indeed, the genomic organization of the leptospiral LPS biosynthesis pathway encompasses up to 30 *rfb* open reading frames for the O antigen, in addition to the *lpx* genes for the lipid A biosynthesis (Pena-Moctezuma et al., 1999; Ren et al., 2003).

The leptospiral genome encodes for more than 170 putative lipoproteins (Haake and Zückert, 2015). Proteomics approaches showed that they are expressed from 1,500 to 35,000 copies/cell (Malmström et al., 2009), illustrating that they are a very abundant component of the bacteria. The main lipoproteins are as follows (in order of relative abundance): LipL32, LipL21, LipL41, LipL36, LipL45, and Loa22 (Malmström et al., 2009). Most of these lipoproteins are conserved in the pathogenic species and serotypes of *Leptospira (* Haake et al., 2000; Cullen et al., 2003); however, only Loa22 has been described as a virulence factor (Ristow et al., 2007).

The two leptospiral endoflagella (EFs) are embedded within the periplasmic space and do not protrude outside of the bacteria. They are inserted at each pole of the bacteria and promote the leptospiral motility by alternating clockwise and counterclockwise rotations. Interestingly, unlike other bacteria such as *Salmonella*, the leptospiral flagellar filament is composed of different flagellin-like proteins: two FlaAs (1-2), four FlaBs (1-4), and two flagella-associated proteins FcpA and FcpB (Gibson et al., 2020). The complex structure of the leptospiral flagellum was recently resolved for *L. biflexa* and evidenced an asymmetrical assembly in which FlaBs form the core of the flagellar filament, enveloped by all FlaAs and Fcps in a structure called sheath (Gibson et al., 2020) (**Figure 2**). These EFs hence differ largely from the classical flagella structure, described in *Salmonella*, and in which one type of monomer, FliC, assembles to form 11 protofilaments forming the flagellar filament. Additionally, the FlaB subunits, which compose the core of the EFs, have been predicted to be structurally different from FliC. Indeed, they would possess the D0–D1 domains but would lack the immunogenic D2–D3 domains (Holzapfel et al., 2020) (**Figure 2**).

PG is an essential component of the cell wall that protects bacteria from turgor pressure (Vollmer et al., 2008). Although the specific components vary between Gram- and Gram+ bacteria, PG is always composed of alternating sugars cross-linked by various peptides. Linear chains made of a repetition of N-acetyl-glucosamine (GlcNAc) and N-acetyl-muramic acid (MurNAc) are connected by stem peptides bound to the MurNAc sugars. In the case of *Leptospira*, these peptides are **(i)** L-alanine (L-Ala), **(ii)** D-glutamine (D-Glu), **(iii)** meso-diaminopimelic acid (mDAP), and **(iv)** D-alanine (D-Ala) (Cameron, 2015) (**Figure 2**). The cross-linking occurs between mDAP in position 3 and D-Ala in position 4. One peculiarity of the leptospiral PG is that it is located close to the inner membrane (Cameron, 2015). The outer membrane is therefore more fluid, as it is not anchored to the PG like in classical Gram negative bacteria (Charon et al., 1981; Raddi et al., 2012). Furthermore, the leptospiral PG is responsible for the helix shape of the bacteria (Slamti et al., 2011).

### Innate Immunity and PRRs—Toll-like Receptors, NODs and NOD-like Receptors

Cells of the mammalian innate immune system play a key role in sensing pathogens upon infection. Sensing relies on the expression of germline-encoded conserved receptors, called pattern recognition receptors (PRRs) that recognize microorganism-specific patterns. These microbe-associated molecular patterns (MAMPs) are components that have been highly conserved throughout evolution and that are essential to microbial survival but absent from the host.

**Figure 3:**
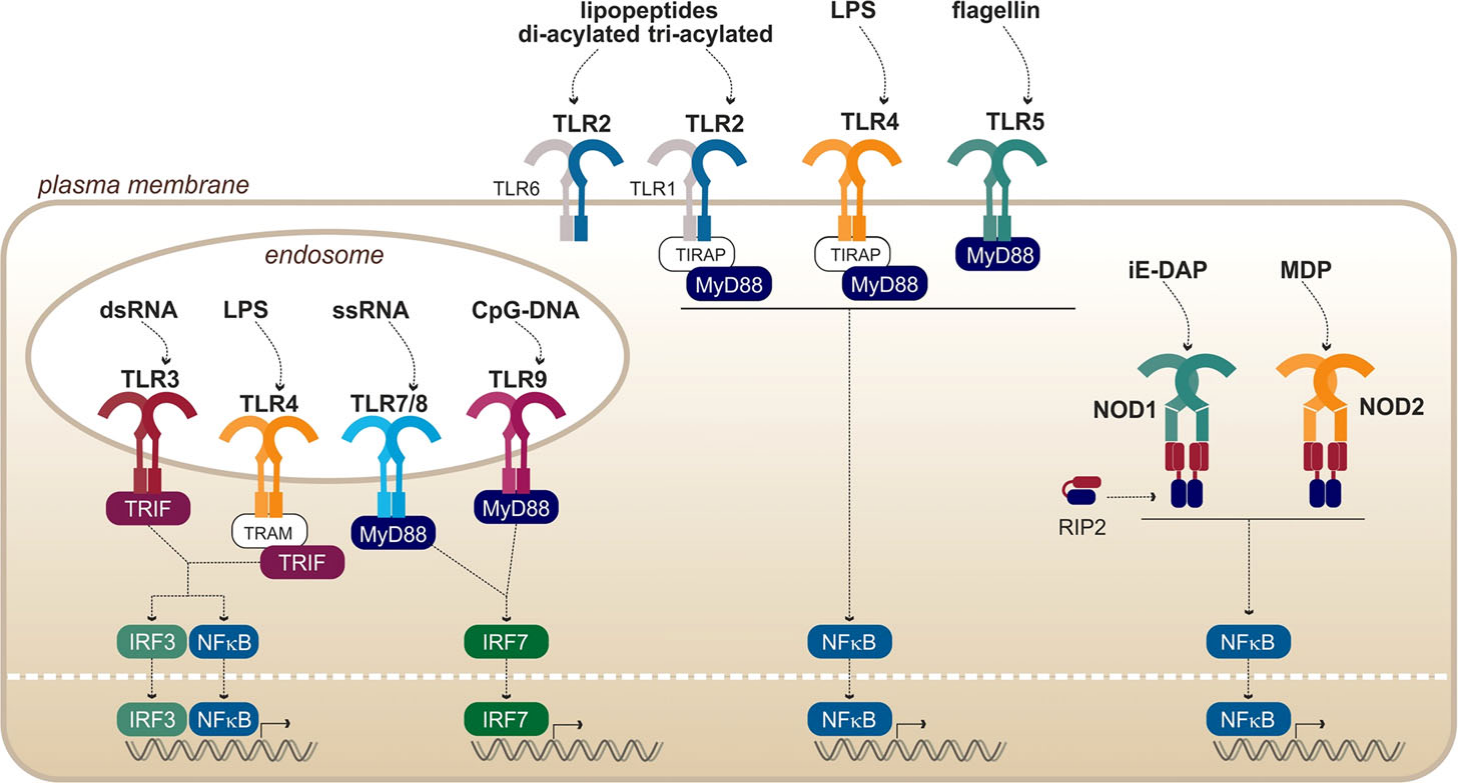
Overview of PRRs from the TLRs and NODs families, with their respective ligands. Adapted from Kawai and Akira (2010).

Toll-like receptors (TLRs) are eukaryotic transmembrane receptors expressed in immune (myeloid and lymphoid) and non-immune (fibroblast, epithelial, and endothelial) cells, which recognize a large variety of microbial ligands (**Figure 3**). TLRs are proteins composed of 750–1,000 residues and are ~ 75–100 kDa. The structure of TLRs reveals that they are transmembrane proteins composed of **(i)** an N-terminal extracellular domain for ligand binding composed of hydrophobic leucine-rich repeats (LRR), forming a horseshoe structure; **(ii)** one transmembrane helix of ~ 20 hydrophobic residues; and **(iii)** a C-terminal cytosolic toll/interleukin-1 receptor domain for signal transduction (Botos et al., 2011). Upon binding of the ligand through the extracellular domain, TLRs dimerize to form either homo- or heterodimers. Once the dimers are formed, the intracellular TIR domains of the two TLRs interact and activate cytosolic adaptors to induce signaling cascades leading to inflammatory and antimicrobial responses. It is interesting to note that the exchange of one amino acid within the ligand-binding domain of a TLR or its co-factor can alter its specificity (Meng et al., 2010; Osvaldova et al., 2014; Lozano-Aponte et al., 2020).

In addition to the membrane-bound TLRs, nucleotide-binding oligomerization domain (NOD) receptors and NOD-like receptors (NLRs) are sensors that are present in the cytosol of immune and epithelial cells. They recognize, among others, fragments of bacterial peptidoglycan (**Figure 3**), thus providing a redundancy of the pathogen recognition system. The first common feature of all NODs and NLRs is their conserved central NOD oligomerization domain. Furthermore, most of them harbor a hydrophobic LRR domain in the C-terminal portion allowing ligand binding. The signal transducing N-terminal domains are however diverse and are the basis of the NLR classification (Fritz et al., 2006; Carneiro et al., 2007).

### Relationship Between Host-Specificity of TLR and NOD Responses Against *Leptospira* and Multifaceted Leptospirosis

Leptospires are pathogenic bacteria that can infect vertebrates, and the disease presents itself in several forms depending on the hosts. Nevertheless, the immune mechanisms that determine the severity of the infection and that confer susceptibility to leptospirosis remain enigmatic. Early recognition of microbes by innate immune receptors plays a key role in the onset of an infection. Therefore, we hypothesize that differential escape/recognition of leptospires by PRRs of various hosts could influence the outcome of the disease and understanding these mechanisms could be key to shedding light on the host-specificity of leptospirosis. In this review, we will summarize the current knowledge on leptospire recognition by PRRs of the TLR, NOD, and NLR families in human, mouse, bovine, and canine. First, we will describe the responses that seem to occur independently of the host species, namely TLR2 and NLRP3. Then, we will focus on other responses, for which host-specificities have been described: NOD1, TLR5, and most importantly TLR4.

## Conserved recognition—in both resistant and susceptible hosts

Upon infection with *Leptospira interrogans*, the activation of some PRRs has been shown to be conserved in both resistant and susceptible hosts. These conserved activation responses include the activation of TLR2 by the leptospiral lipoproteins and the activation of the NLRP3 inflammasome. Both of these will be discussed in this section.

### TLR2 and Leptospiral Lipoproteins

TLR2 is the receptor that allows the recognition of lipopeptides such as bacterial lipoproteins, lipoteichoic acids (from Gram+ bacteria), and lipomannans (from mycobacteria). TLR2 is known to heterodimerize with either TLR1 to accommodate tri-acylated lipopeptides present in Gram bacteria or TLR6 for di-acylated lipopeptides found in Gram+ bacteria.

#### Activation Mechanism

The structures of these dimers with their respective synthetic ligands Pam_3_CSK_4_ and Pam_2_CSK_4_ have been solved (Jin and Lee, 2008; Kang et al., 2009) and showed one ligand binding per dimer. The structural analysis of the TLR2/TLR1 heterodimer revealed that both TLRs have a hydrophobic pocket on the convex side of their LRR domain at the interface between the central and C-terminal sections (LRR10–LRR15), hence providing unusual binding sites for lipidic anchors (Jin and Lee, 2008). It was shown that two lipidic anchors of the Pam_3_CSK_4_ ligand interact with TLR2, whereas the third one interacts with TLR1 (Jin and Lee, 2008). In the case of the TLR2/TLR6 heterodimer, it was shown that only TLR2 contributes to Pam_2_CSK_4_ lipidic chain support (Kang et al., 2009). Indeed, sequence analyses showed that despite a 56% sequence homology with TLR1, TLR6 does not have the hydrophobic residues essential for lipid accommodation (Jin and Lee, 2008; Botos et al., 2011). However, it was suggested that TLR6 could interact with the peptide fragment directly (Kang et al., 2009). Furthermore, for both sets of heterodimers, it has been suggested that weakly bound heterodimers are constitutively preformed at the plasma membrane and that the ligand binding induces the rearrangement of these dimers into actively signaling platforms.

#### Host-Specificity of TLR2 Activation

TLR2 signaling upon ligand binding is well conserved in different host species (Werling et al., 2009), with the particularity that for avian TLR2, only one heterodimer is present and allows recognition of both di- and tri-acylated peptides (Keestra et al., 2007; Werling et al., 2009). Additionally, a study on wild brown rats (*Rattus norvegicus*) in China reported little intraspecies diversity for TLR2 and reported few polymorphism sites, compared to other TLRs such as TLR8 (Su et al., 2021). Of note, the murine (m)TLR2 hydrophobic binding site is shorter than that of the human (h)TLR2, hence resulting in a better accommodation of very short lipopeptides (Botos et al., 2011).

#### Leptospiral Recognition by TLR2

Leptospires are potent agonists of human, mouse, and canine TLR2 (Werts et al., 2001; Nahori et al., 2005; Hsu et al., 2010; Novak et al., 2022). Furthermore, in mice, TLR2 activation is dependent on TLR2/1 heterodimer formation (Nahori et al., 2005), leading to the recognition of tri-acylated lipoproteins. Another study reported TLR2-dependent activation of a bovine fibroblast cell line (Guo et al., 2016), suggesting that the bovine receptor could also be activated. The same group also suggested that porcine TLR2 could be activated in response to *Leptospira* infection (Guo et al., 2015). LipL32, the major lipoprotein of leptospires, has been demonstrated to be a TLR2 ligand (Werts et al., 2001; Hsu et al., 2010), activating the receptor through hydrophobic interactions (Hsu et al., 2010; Hsu et al., 2017). Furthermore, a calcium-binding cluster on LipL32 is essential to sustaining the lipoprotein structure and allows proper TLR2 signaling (Lo et al., 2013). Loa22 has also been shown to be a ligand for TLR2 (Hsu et al., 2021), and several lipopeptides from leptospiral outer membrane proteins (OMPs) have been predicted to interact with TLR2 (Akino Mercy and Natarajaseenivasan, 2021). However, these are probably not the only lipoproteins that signal through TLR2, although initial results reported that LipL41 does not stimulate TLR2 (Yang et al., 2006). Of note, the TLR2 activity of the leptospiral LPS (Werts et al., 2001) is conferred by co-purifying lipoproteins, among them LipL32, that can be removed only upon extensive purification (Bonhomme et al., 2020).

In addition to the formation of TLR2/1 heterodimers, the recognition of leptospiral lipoproteins also seems to involve the CD14 molecule, a member of the LPS–receptor complex involving CD14, LPS-binding protein (LBP), MD2, and TLR4. However, CD14 is also important for the signaling of some tri-acylated lipoproteins through TLR2/1 complexes (Ranoa et al., 2013). In the case of *Leptospira*, activation of hTLR2 was shown to be CD14-dependent (Werts et al., 2001). Of note, such a mechanism is conserved in other Spirochetes such as *Borrelia* and *Treponema (* Sellati et al., 1998; Wooten et al., 1998; Sellati et al., 1999). Although the proper function of CD14 in TLR2 signaling remains unknown, it is hypothesized that it could contribute to support binding of the hydrophobic chains of the lipoproteins, similar to the mechanism of recognition of lipid A and TLR4 (Ranoa et al., 2013). Interestingly, very little host-specificity was described for CD14 (Delude et al., 1995), suggesting again that TLR2 signaling upon activation by leptospires is conserved between species.

### NLRP3 and Leptospiral Glycolipoprotein

The inflammasome is a multiprotein complex and acts as a cytosolic protein sensor that, upon activation, leads to the cleavage and maturation of key inflammatory cytokines such as IL-1β and IL-18. Among the numerous NLRPs encoded, the NLRP3 inflammasome is the best described and is relevant in the context of infection by *L. interrogans*.

#### NLRP3 Activation Mechanism

In mice, activation of the NLRP3 inflammasome requires the integration of (at least) two signals to be fully functional. First, the NLRP3 system needs to be primed. Such priming occurs by stimulating PRRs with MAMPs or cytokines (such as TNF), followed by the translocation of NF-κB (Bauernfeind et al., 2009; Franchi et al., 2009). This priming allows the transcription of NLRP3 mRNA as well as pro-cytokines mRNA, making the cell more responsive to subsequent activation (Bauernfeind et al., 2009). The second signal leads to the formation of the actual inflammasome complex and requires activation by various cellular stress factors, including mitochondrial reactive oxygen species (ROS) (Lawlor and Vince, 2014) production, calcium (Ca^2+^) influx (Brough et al., 2003), toxins, and lysosomal leakage (He et al., 2016). NLRP3 activation often arises from potassium (K^+^) efflux, caused by damaged or downregulated sodium/potassium pumps (Muñoz-Planillo et al., 2013; He et al., 2016). Upon NLRP3 activation, the ASC adaptor is recruited to the inflammasome and oligomerizes in filaments that serve as amplification platforms for caspase cleavage (Dick et al., 2016). Caspases are cysteine proteases that play an essential role in cell death and immunity. Most caspases function in a similar manner: they are constitutively inactive in pro-caspase form and are cleaved and activated upon stimuli (Yamin et al., 1996). Caspases then cleave substrates with aspartic residues. Caspase 1 was the first caspase to be described as part of the inflammatory response. It carries the interleukin-converting enzyme (ICE) activity and catalyzes mature IL-1β and IL-18 from pro-IL-1β and pro-IL-18 (Miller et al., 1993; Sansonetti et al., 2000).

#### Host-Specificity of NLRP3 Activation

NLRP3 activation has been shown to require different signals for activation in different host species. More specifically, human monocytic cells, such as THP1, are much more sensitive to inflammasome activation and only require one signal such as LPS to trigger potent IL-1β secretion (Netea et al., 2009; Wang et al., 2013). As human monocytes can release ATP, it is hypothesized that this ATP release could act as endogenous signal 2, hence triggering proper NLRP3 activation (Netea et al., 2009; Wang et al., 2013).

#### Leptospiral Activation of NLRP3

**Figure 4:**
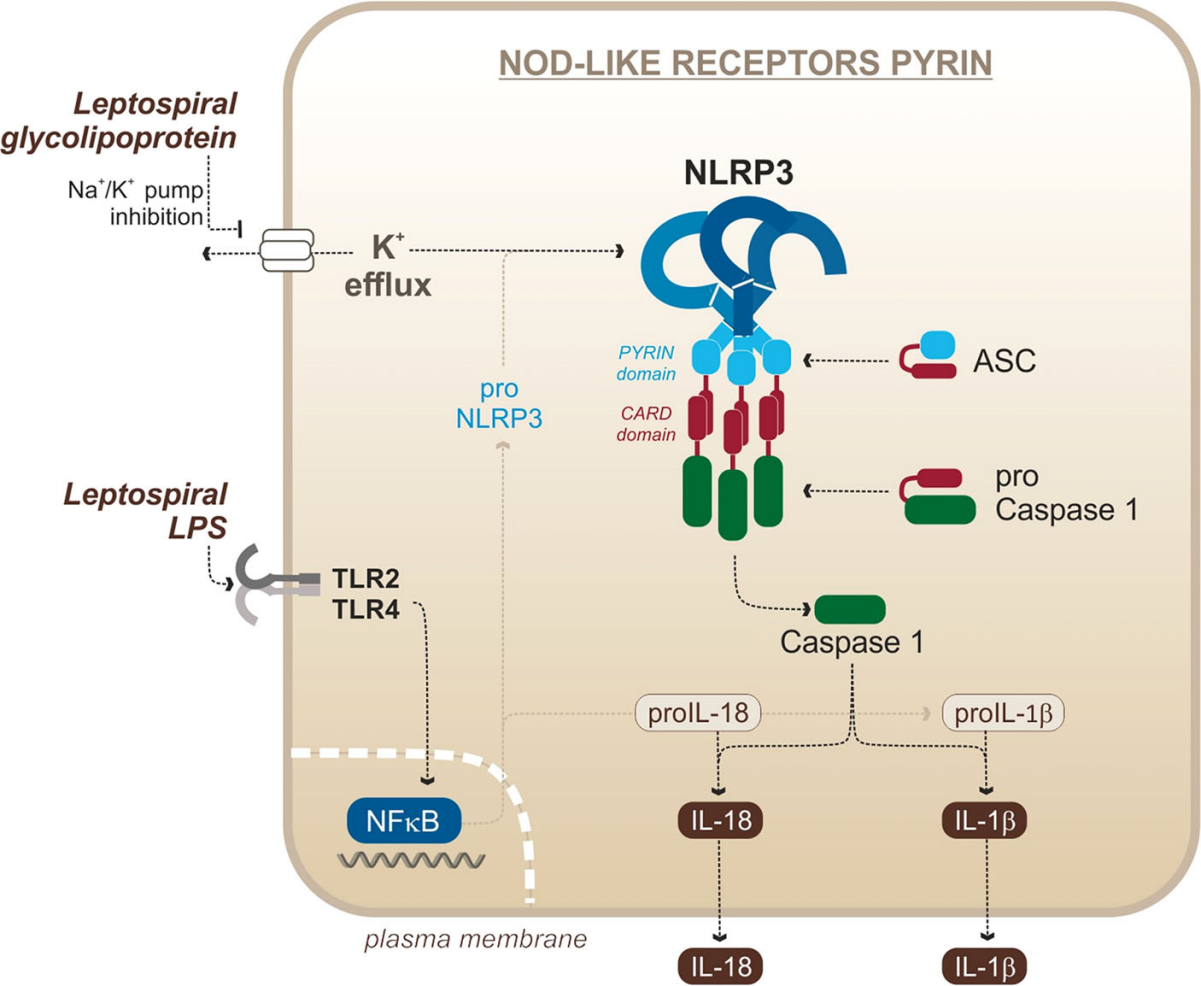
Leptospiral activation of the murine NLRP3 canonical inflammasome. Adapted from Lacroix-Lamande et al. (2012).

In the case of *L. interrogans*, our group published that NLRP3 is activated upon infection of murine macrophages (Lacroix-Lamande et al., 2012) (**Figure 4**). The priming signal is the activation of TLR2/TLR4 by the leptospiral LPS and associated lipoproteins, and signal 2 is the downregulation of the sodium/potassium pump by the leptospiral glycolipoprotein (GLP), provoking a potassium efflux, a classical trigger of the NLRP3 activation (Lacroix-Lamande et al., 2012). As a result of NLRP3 activation and caspase 1 cleavage, leptospires induce a TLR2-TLR4-dependent production of IL-1β which was shown to be independent of ROS production in murine cells (Lacroix-Lamande et al., 2012). These results were confirmed by a study showing a role of doxycycline in the reduction of IL-1β production by the NLRP3 inflammasome (Zhang et al., 2017). Consistent with IL-1β found in blood of leptospirosis patients (Senavirathna et al., 2020), another study showed that IL-1β and IL-18 were also produced in human cells through NLRP3 activation (Li et al., 2018). This study reported that NLRP3 activation in human cells occurs through the production of ROS, suggesting different activation mechanisms in human and murine cells (Li et al., 2018). However, it remains to be confirmed since this study was recently brought into question (Li et al., 2021). In addition, other studies reported IL-1β release upon stimulation of canine whole blood and monocyte-derived dendritic cells (moDCs) in response to Leptospira (Rajeev et al., 2020; Novak et al., 2022), suggesting that functional inflammasomes could also be triggered in dogs, although the mechanism remains uncharacterized. Taken together, these data suggest that leptospires could trigger NLRP3 inflammasome and induce IL-1β secretion in different hosts.

## Efficient escape—with strengthened mechanisms in mice

*Leptospira interrogans* are stealth pathogens that have been shown to escape recognition by some innate immune receptors. In the following section, we specifically discuss those escape mechanisms that shield leptospires from detection *via* TLR5 and NODs. Furthermore, we also discuss that the study of the species-specificity of TLR5 and NOD responses highlights the mechanism of escaping mouse receptors.

### TLR5 and Leptospiral Endoflagella

TLR5 is the receptor responsible for the recognition of the bacterial flagellin: the protein component of the flagella that is essential for bacterial motility (Hayashi et al., 2001). Although the flagellin subunits are oligomerized in the bacteria, TLR5 is only activated upon release of monomeric flagellin subunits (Smith et al., 2003), specifically fliC.

#### TLR5 Activation Mechanism

FliC monomers (the globular proteins constituting the flagellum) from *Salmonella enterica* are divided into four domains (D0, D1, D2, and D3). The structure of the complex showed that TLR5 interacts with FliC in the D1 domain *via* the side of its LRR domain at the interface between the N-terminal and the central section (LRR-NT–LRR10) (Yoon et al., 2012). Previous mutagenesis analyses in *E. coli* had already implicated the D1 domain in TLR5 activation (Donnelly and Steiner, 2002). Upon ligand binding, two TLR5/FliC complexes then form an active homodimer that get stabilized by the interaction of each FliC D0 domain with the convex side of the opposite TLR5 (Yoon et al., 2012).

#### Host-Specificity of TLR5 Activation

The murine TLR5 is less stringent than its human counterpart, and it accommodates more diverse substrates (Andersen-Nissen et al., 2007; Forstnerič et al., 2016). Canine and avian TLR5 were also reported to be functional and to respond to *Salmonella* flagellin (Keestra and de Zoete, 2008; Zhu et al., 2020). On the contrary, bovine and porcine TLR5 is much harder to activate than human TLR5 and seems to have poor flagellin sensing ability against the fliC components tested (Metcalfe et al., 2014). Indeed, neither of them seem to recognize flagellin from *Salmonella (* Metcalfe et al., 2014; Faber, 2018). A study reported that replacement of two amino acids in the TIR domain of the bovine TLR5 could be responsible for at least the partial lack of responsiveness, compared to human TLR5 (Osvaldova et al., 2014).

#### Leptospiral Escape from TLR5

**Figure 5:**
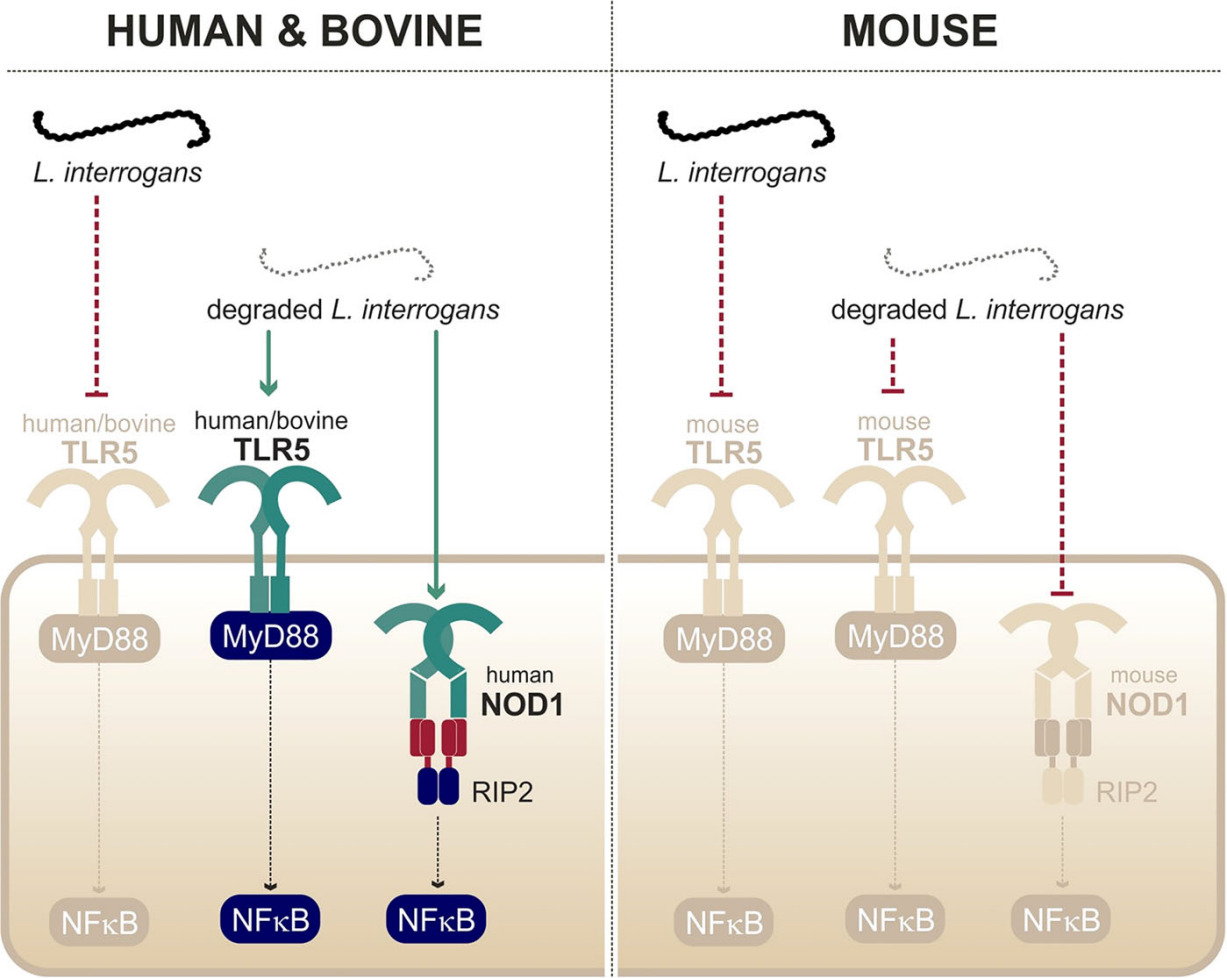
Recognition of degraded leptospires only by human/bovine TLR5 and human NOD1. Adapted from Ratet et al. (2017) and Holzapfel et al. (2020).

A study from our group showed that live leptospires largely escape TLR5 recognition through an efficient hiding of the agonists, presumably due the peculiar structure and localization of the leptospiral periplasmic flagella (Holzapfel et al., 2020). This was further supported by the findings that the course of leptospirosis is unaltered in TLR5^-/-^ mice (Holzapfel et al., 2020). Interestingly, it was shown that heat treatment allowed triggering of TLR5 signaling by degraded leptospires (Holzapfel et al., 2020) (**Figure 5**). Such findings were very recently confirmed by another group (Novak et al., 2022). Leptospiral flagellin monomers, supposedly TLR5 agonists, were unexpectedly released only after boiling for 30 min and have been shown to be unusually thermoresistant in comparison to *Salmonella* FliC monomers (Holzapfel et al., 2020). The study of the species-specificity of TLR5 requires methodological caution as the heterologous expression in HEK293T cells might be biased by the endogenous expression of human TLR5 in these cells. A recent study performed in these cells suggested no difference between human TLR5 and mouse TLR5 activation by degraded leptospires (Novak et al., 2022), whereas a heterologous expression of the different TLR5 vectors in specific HEK293T-hTLR5 knockdown cells demonstrated species-specificity (Holzapfel et al., 2020). Indeed, we found that both human and bovine TLR5 recognize heat-killed leptospires, although the mouse TLR5 did not sense the Icterohaemorrhagiae and Manilae serovars but could scantily recognize the Copenhageni serovar (Holzapfel et al., 2020). These results were unexpected considering the low stringency of the murine receptor and very interestingly showed that the specificity of the leptospiral recognition by TLR5 is not the same as for *Salmonella*. We hypothesize that the lack of D2/D3 domains in the leptospiral FlaB subunits could play a role in the interaction with human and bovine TLR5. Although these domains traditionally stabilize the murine TLR5 homodimer (Yoon et al., 2012), they could play an opposite role with the bovine TLR5 in which the ligand-binding region was shown to be mutated (Tahoun et al., 2017). Importantly, our group also showed that antimicrobial peptides were active against live bacteria to allow for the signaling through human and bovine TLR5 (Holzapfel et al., 2020), most probably through membrane disruption. Overall, these data show that live leptospires escape efficiently TLR5 recognition in human, bovine, and murine hosts. Furthermore, an additional escape mechanism seems to prevent the recognition of the released leptospiral TLR5 agonists by the murine receptor, suggesting a strengthened escape mechanism in mice.

### NOD1/NOD2 and Leptospiral Peptidoglycan

NOD receptors, namely, NOD1 and NOD2, are cytosolic sensors that become activated upon sensing of bacterial peptidoglycan (PG) fragments, called muropeptides.

#### NOD Activation Mechanism

NOD1 recognize γ-glutamyl diaminopimelic acid (iE-DAP), and this recognition is increased when iE-DAP is linked to the sugar part, forming muramyl tripeptide (MTP), a fragment mostly present in Gram- bacteria. On the other hand, NOD2 recognizes muramyl dipeptide (MDP) that is present in both Gram+ and Gram- bacteria (Girardin et al., 2003) (**Figure 5**). The caspase recruitment domain (CARD) of NODs functions like the TIR domain of TLRs and leads to homotypic interactions between NODs and their adaptor RIP2 that bridges downstream signaling (Bertin et al., 1999; Kobayashi et al., 2002). RIP2 leads (*via* TAK1, IKK kinases, and NEMO) to the degradation of IκB, hence promoting NF-κB translocation in the nucleus, allowing inflammatory responses.

#### Host-Specificity of NOD Activation

Study of the species-specificity of NOD1 has demonstrated that human and mouse NOD1 do not respond to the same fragments of bacterial peptidoglycan. Indeed, mouse NOD1 recognizes mainly muramyl tetra-peptide whereas human NOD1 preferentially senses muramyl tri-peptide (Magalhaes et al., 2005). This host-specificity is most likely attributable to different key residues (around amino acids 816 and 844) in the ligand-binding LRR domain of NOD1 (Girardin et al., 2005). To the best of our knowledge, no host-specificity has been shown for NOD2.

#### Leptospiral Escape from NODs

In contrast to other bacteria in which structural modifications of muropeptides impair recognition through NOD1 and NOD2, our group found that *Leptospira* escape both receptors by a novel mechanism. Indeed, LipL21, one of the major leptospiral lipoproteins, tightly binds to the leptospiral PG, thus preventing muropeptide release and recognition (Ratet et al., 2017). Therefore, NOD1 and NOD2 play no role in controlling *Leptospira* dissemination *in vivo*. However, upon bacterial degradation and extensive purification of the leptospiral PG (4-h boiling protocol to remove LipL21), an agonist stimulating human NOD1 but barely human NOD2 was released (**Figure 5**). Consistently, it was further shown that muramyl tri-peptide, the agonist of human NOD1, is present in the purified leptospiral PG (Ratet et al., 2017). Interestingly, the purified leptospiral PG is devoid of muramyl tetra-peptides, usually found in Gram- bacteria, and can therefore not be sensed by mouse NOD1, even after extensive purification (Ratet et al., 2017). Overall, the leptospiral PG, in the context of live bacteria, is not recognized by the NOD receptors in either human or murine host, thanks to its tight association with LipL21. Furthermore, in the murine host, leptospires have redundant mechanisms with both the LipL21 phenotype and the absence of the NOD1 ligand (Ratet et al., 2017), suggesting again a reinforced escape mechanism in mouse.

## Species-specific recognition

### TLR4 and Leptospiral LPS

TLR4 was the first TLR to be identified in mammals and was described as the receptor of bacterial lipopolysaccharides (LPS) (Medzhitov et al., 1997; Poltorak, 1998). Contrary to other TLRs, TLR4 activation involves numerous cofactors that participate in LPS recognition, including CD14, LBP, and MD2.

#### TLR4 Activation Mechanism

TLR4 is activated upon binding with the lipid A moiety of the LPS. However, the accommodation of the LPS requires several steps and cofactors before ligand-induced dimerization of TLR4, allowing downstream signaling. First, in the circulation, aggregated LPS molecules bind to a protein called LPS-binding protein (LBP). One LBP molecule can interact with LPS micelles through its N-terminal domain to transfer individual molecules of LPS to the next partner, CD14 (Wright et al., 1990; Hailman et al., 1994; Ryu et al., 2017). CD14 then binds transiently to the LPS/LBP complexes to receive one LPS molecule (Wright et al., 1990; Frey, 1992; Hailman et al., 1994; Ryu et al., 2017). Finally, CD14 transfers the LPS to the myeloid differentiation factor 2 (MD2)/TLR4 complex that is the final receptor of LPS. Upon activation, TLR4 triggers the activation of both adaptors MyD88 at the plasma membrane and TRIF upon internalization in endosomes.

#### Host-Specificity of TLR4 Activation

The affinity of the MD2/TLR4 complex for lipid A directly determines the activation level in different host species (Anwar et al., 2015). Two main features of lipid A have been described to alter the affinity for the MD2/TLR4 complex. First, the number of acyl chains on lipid A is important. Indeed, the complex is most efficient when dealing with hexa-acylated lipid A such as the one from *E. coli.* Penta-acylated lipid A is poorly recognized, and tetra-acylated lipid A can even act as an antagonistic molecule of the human complex (Rietschel et al., 1994; Teghanemt et al., 2005). Second, lipid A usually presents two phosphoryl groups on its disaccharide (positions 1 and 4′) that increase affinity with the MD2/TLR4 complex by interacting with positively charged residues on TLR4 (Rietschel et al., 1994; Park et al., 2009). Interestingly, human, equine, and canine TLR4 have partial charges in the binding sites for the lipid A phosphate groups, whereas murine TLR4 has full charges, allowing easier ligand accommodation (Lozano-Aponte et al., 2020). Overall, the murine TLR4 seems to be less stringent that its counterparts from other species.

#### Leptospiral Escape from TLR4 Recognition

**Figure 6:**
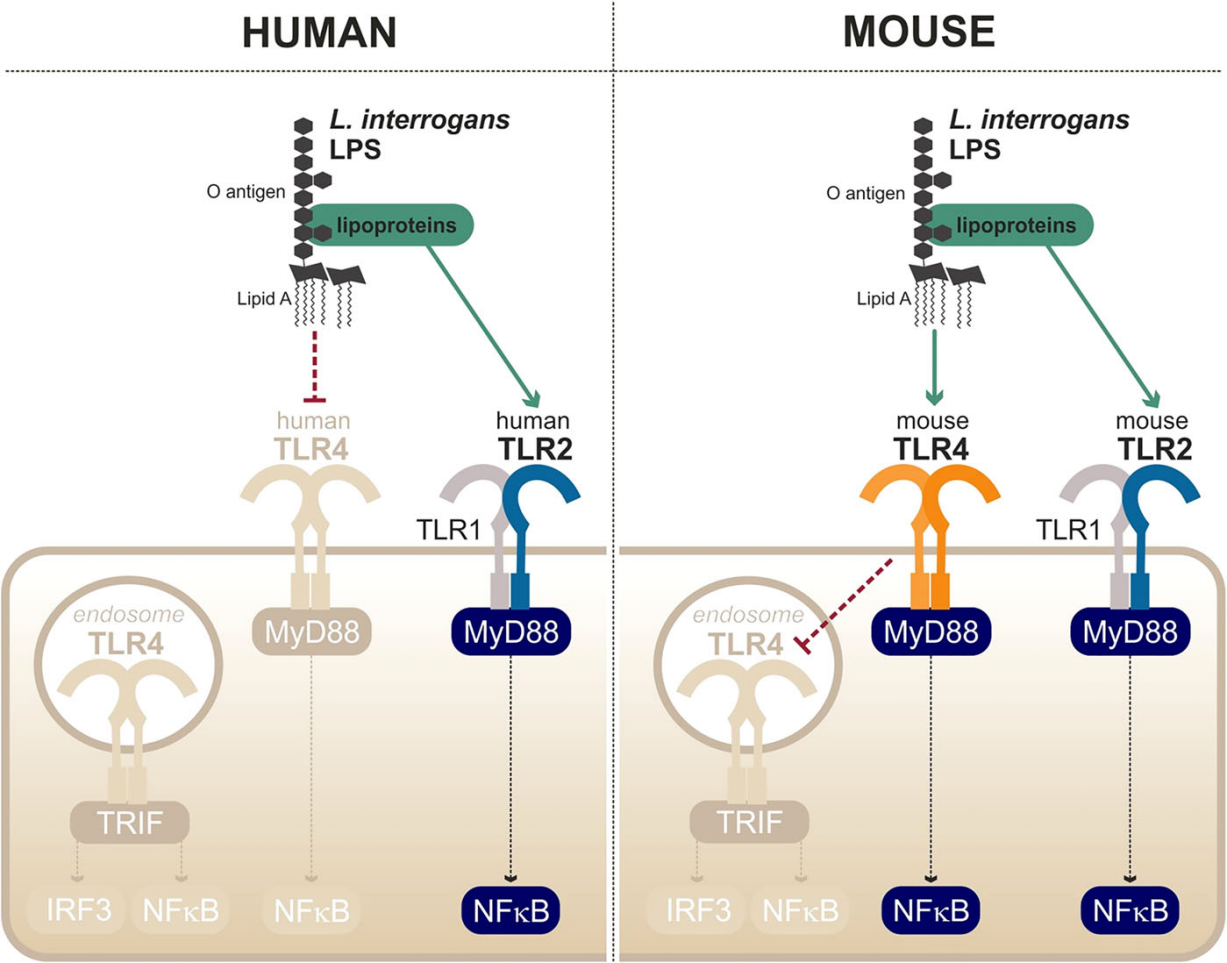
Escape and partial recognition of the leptospiral LPS by human and mouse TLR4. Adapted from Werts et al. (2001), Nahori et al. (2005), Bonhomme et al. (2020).

The first peculiarity of the leptospiral lipid A is that it is recognized by the murine TLR4 but not by the human TLR4 (Nahori et al., 2005) (**Figure 6**). It is hypothesized that the methylated 1-phosphate and missing 4′-phosphate groups are the cause of the lack of recognition by human TLR4. Likewise, the leptospiral LPS reacts poorly to the traditional endotoxin quantification limulus amebocyte lysate (LAL) test (Nahori et al., 2005). The very low endotoxicity of the leptospiral LPS makes studies on reporter systems very sensitive to any other endotoxin contaminations, for instance from bacterial culture medium EMJH or cell culture products that are often contaminated with residual endotoxin. Such problems could account for the discrepant results in the literature that artefactually suggest that the leptospiral LPS could activate human TLR4 (Novak et al., 2022).

Interestingly, data from our group have shown that the leptospiral LPS remains poorly endotoxic, even on murine TLR4, compared with classical LPS from *E. coli* or *S. enterica (* Nahori et al., 2005). We recently published a study showing that the leptospiral LPS escapes the internalization of TLR4 in murine macrophages (Bonhomme et al., 2020), consequently avoiding the activation of TRIF-dependent responses such as NO, IFN-γ, and RANTES (Bonhomme et al., 2020) (**Figure 6**). The phenotype was shown to be dependent on the leptospiral O antigen and the presence of copurifying lipoproteins (Bonhomme et al., 2020). More interestingly however is the fact that leptospiral LPS escapes human TLR4 completely, whereas it only partially escapes murine TLR4 recognition/activation, consistent with the lower stringency of the mouse receptor.

Finally, the last particularity of the leptospiral LPS is its ability to activate TLR2 in both human and murine cells (Werts et al., 2001) (**Figure 6**). Although it was initially believed that the observed TLR2 activity could be linked to the LPS itself, it is in fact independent of the leptospiral lipid A and rather due to contaminating lipoproteins, consistent with the TLR2/TLR1 response (Nahori et al., 2005). As TLR2 responses to leptospiral lipoproteins are not species-specific, the contaminating TLR2 activity of the leptospiral is conserved in different hosts (Werts et al., 2001).

## Discussion

**Figure 7:**
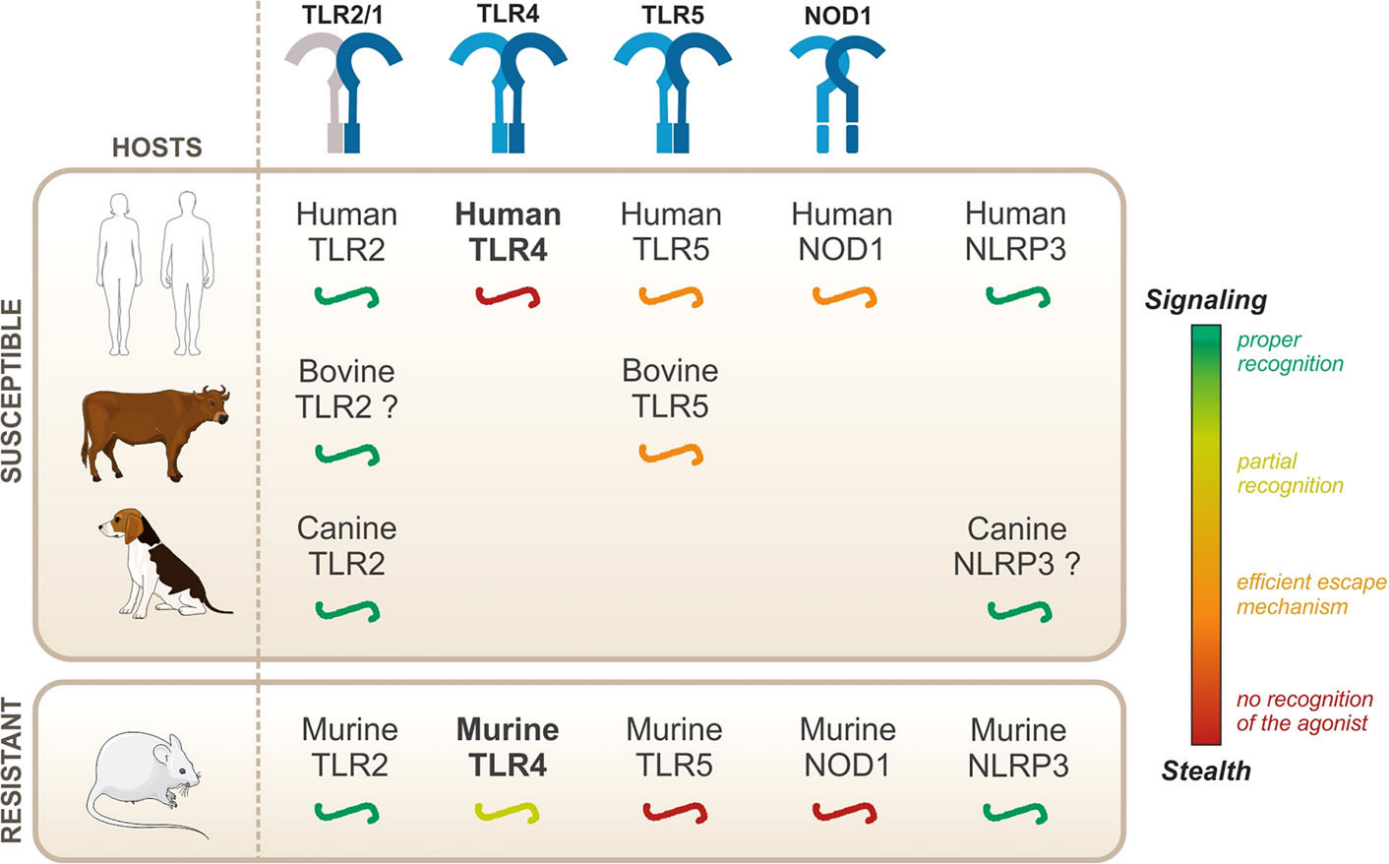
Overview of the species-specificity of PRR recognition of leptospires.

As mentioned previously, leptospires can infect all mammals, but leptospirosis symptoms vary according to the hosts. They are therefore classified as either resistant or susceptible, depending on whether they might present acute symptoms of leptospirosis. However, to date, the innate immune mechanisms underlying such resistance or susceptibility remain unknown. In this review, we have summarized the current knowledge on leptospiral recognition by PRRs of the TLR, NOD, and NLR families (**Figure 7**).

Leptospires are potent TLR2 agonists, through the recognition of their tri-acylated lipoproteins by TLR1/TLR2, which was shown to be conserved in both humans and mice (Werts et al., 2001; Nahori et al., 2005). Similarly, leptospires activate NLRP3 in different hosts (Lacroix-Lamande et al., 2012; Li et al., 2018). Given these results, although they are essential for the proper recognition of the leptospires, it is unlikely that TLR2 and NLRP3 could mediate the species-specificity of the leptospiral recognition. Second, live leptospires escape TLR5 and NODs in different hosts, both susceptible and resistant, suggesting that there is no major species-specificity for these responses. It was further demonstrated that, despite efficient escape mechanisms, artificial release of the agonists revealed human/bovine TLR5 and human NOD1 activities, but not murine TLR5 nor murine NOD1 activities (Ratet et al., 2017; Holzapfel et al., 2020). Human/bovine TLR5 and human NOD1 might therefore play a specific role during acute leptospirosis in a susceptible host, considering that they can sense released agonists upon bacterial degradation. Whether their contribution could be beneficial to human and bovine hosts, potentially to compensate for the lack of TLR4 activation in these hosts, remains to be addressed.

The most interesting mechanism in the species-specific recognition of *Leptospira* is the peculiar sensing of its LPS by TLR4. The role of TLR4 in the resistance of the mouse model has been extensively addressed: C3H/HeJ and TLR4^-/-^ mice are sensitive to leptospirosis (Pereira et al., 1998; Nally et al., 2005; Viriyakosol et al., 2006; Chassin et al., 2009). Our results further showed that the recognition by the murine TLR4 was only partial, because of the escape of TLR4 internalization and subsequent TLR4-TRIF escape (Bonhomme et al., 2020), potentially contributing to the chronicity of the infection in mice. TLR4-MyD88 activation by the leptospiral LPS therefore remains the best candidate to explain the species-specificity of the immune response to *Leptospira*. However, a recent study demonstrated that transgenic mice expressing human TLR4/MD2 are not more susceptible to leptospirosis than WT mice (Nair et al., 2021). This study concludes that the presence of a functional TLR4 gene, whether murine or human, is the prerequisite for resistance to acute leptospirosis (Nair et al., 2021). Of note, this study did not use mice transgenic for human CD14, most probably because very little species-specificity has been described for CD14 (Delude et al., 1995). Unexpectedly, these findings could shed light on the fact that most hosts from susceptible species, such as 90% of humans, do not develop symptoms of acute illness (Costa et al., 2015). It is therefore proposed that TLR4 is essential to the resistance of the murine model, but the lack of recognition by human TLR4 is not the main reason for the enhanced human susceptibility to leptospirosis. Hence, the precise mechanisms by which TLR4 confers the resistance to the mouse model remain to be determined. TLR4 therefore plays an unequivocal role in the response to leptospirosis. However, host species-specificity seems more complex than the straightforward hypotheses we initially favored. Conciliating all the results of the current literature inevitably requires the involvement of other species-specific mechanisms that are still to be identified. Indeed, the role of TLR4 is not limited to LPS sensing. For instance, TLR4 is known to play an important role in homeostatic immunity and has been shown to be instrumental for the constitutive production by B1 cells of natural IgM, which recognize varied phospholipid and carbohydrate motifs (Panda and Ding, 2015; Dyevoich et al., 2020). Interestingly, such B1-produced antibodies were shown to be essential to the control of relapsing borreliosis, caused by spirochetes *Borrelia (* Belperron and Bockenstedt, 2001; Malkiel et al., 2009). Our group showed in the murine model of leptospirosis that TLR4-dependent IgM produced as early as 3 days postinfection was partially protective (Chassin et al., 2009). Overall, further investigation will be necessary to clarify the role of TLR4 in controlling leptospirosis.

The relative expression of different TLRs in various organs of different host species could also contribute to the species-specificity of the response to *Leptospira*. For instance, it was shown that TLR5 is not expressed in the proximal tubules of mice (Bens et al., 2014), where leptospires chronically reside, hence adding another escape mechanism for the bacteria. Another example is TLR2/TLR1 expression in sheep that seems to be very low in different organs (lung, kidneys, skin) and that could contribute to the species sensitivity (Nalubamba et al., 2007). Another study on female rabbits reported many differences in TLR expression in various organs and reported that TLR expression was low in spleen compared to other organs (Chen et al., 2014). Finally, the cellular composition may also vary from one host to the next. For instance, cattle have a specific subset of macrophages that can produce extracellular traps (bMETs) in response to *Leptospira* (Nagel et al., 2019), a feature that is traditionally associated with neutrophils. Overall, although the cellular composition and differential expression of PRRs are not exhaustively presented in this review, it must be considered when studying immune responses to leptospires.

Finally, the specificities of the numerous leptospiral serovars could also play a role in the species-specificity of the host response, favoring a coevolution of host and pathogen. Indeed, a specific *L. interrogans* serovar Autumnalis strain, in which LPS is deprived of contaminating TLR2 activity, induces self-resolving leptospirosis in mice (Xia et al., 2017). This illustrates that the structural peculiarities of the different serovars, especially on the LPS, might affect greatly the course of the infection. Furthermore, some serovars are strongly associated with hosts, such as serovar Hardjo-Bovis and Pomona that are mostly found in cattle (Adler, 2015; Nagel et al., 2019) and serovar Canicola that is often isolated from dogs (Adler, 2015). Similarly, organ tropisms of the different strains could contribute to the species-specificity of the innate responses to leptospires.

Overall, the species-specificity of the host immune responses to *L. interrogans* remains enigmatic. Studies on TLR2, NLRP3, TLR5, and NODs did not evidence major species-specific responses, at least in responses to live bacteria, reinforcing the hypothesis that TLR4 plays a complex yet instrumental role in host specific responses to *L. interrogans.* However, we believe that the complexity of host specificities in leptospirosis results from the integration of several innate immune mechanisms, which may explain the vast diversity of leptospirosis diseases in different host species.

## Author Contributions

DB wrote the original manuscript and did the figures. CW supervised and edited the manuscript. All authors contributed to the article and approved the submitted version.

## Funding

This work has been funded by Institut Pasteur and Université de Paris Cité through Doctoral school “FIRE” (ED474).

## Conflict of Interest

The authors declare that the research was conducted in the absence of any commercial or financial relationships that could be construed as a potential conflict of interest.

## Publisher’s Note

All claims expressed in this article are solely those of the authors and do not necessarily represent those of their affiliated organizations, or those of the publisher, the editors and the reviewers. Any product that may be evaluated in this article, or claim that may be made by its manufacturer, is not guaranteed or endorsed by the publisher.

## References

[B1] Ackermann K. Kenngott R. Settles M. Gerhards H. Maierl J. Wollanke B. (2021). *In Vivo* Biofilm Formation of Pathogenic Leptospira Spp. In the Vitreous Humor of Horses With Recurrent Uveitis. Microorganisms. 9, 1915. doi: 10.3390/microorganisms9091915 34576809 PMC8464839

[B2] Adler B. (Ed.) (2015). Leptospira and Leptospirosis (Berlin, Heidelberg: Springer Berlin Heidelberg). doi: 10.1007/978-3-662-45059-8

[B3] Akino Mercy C. S. Natarajaseenivasan K. (2021). Htlr2 Interacting Peptides of Pathogenic Leptospiral Outer Membrane Proteins. Microbial Pathogenesis. 155, 104895. doi: 10.1016/j.micpath.2021.104895 33878396

[B4] Andersen-Nissen E. Smith K. D. Bonneau R. Strong R. K. Aderem A. (2007). A Conserved Surface on Toll-Like Receptor 5 Recognizes Bacterial Flagellin. A RT I C L E. 204, 11. doi: 10.1084/jem.20061400 PMC2118731 17283206

[B5] Anwar M. A. Panneerselvam S. Shah M. Choi S. (2015). Insights Into the Species-Specific TLR4 Signaling Mechanism in Response to Rhodobacter Sphaeroides Lipid A Detection. Sci. Rep. 5, 7657. doi: 10.1038/srep07657 25563849 PMC4288214

[B6] Bauernfeind F. G. Horvath G. Stutz A. Alnemri E. S. MacDonald K. Speert D.. (2009). Cutting Edge: NF-κb Activating Pattern Recognition and Cytokine Receptors License NLRP3 Inflammasome Activation by Regulating NLRP3 Expression. J. Immunol. 183, 787–791. doi: 10.4049/jimmunol.0901363 19570822 PMC2824855

[B7] Belperron A. A. Bockenstedt L. K. (2001). Natural Antibody Affects Survival of the Spirochete *Borrelia Burgdorferi* Within Feeding Ticks.Infect. Immun. 69, 6456–6462. doi: 10.1128/IAI.69.10.6456-6462.2001 11553590 PMC98781

[B8] Bens M. Vimont S. Ben Mkaddem S. Chassin C. Goujon J.-M. Balloy V.. (2014). Flagellin/TLR5 Signalling Activates Renal Collecting Duct Cells and Facilitates Invasion and Cellular Translocation of Uropathogenic *E Scherichia Coli*: TLR5 Signalling in Renal Collecting Duct Cells. Cell Microbiol. 16, 1503–1517. doi: 10.1111/cmi.12306 24779433

[B9] Bertin J. Nir W.-J. Fischer C. M. Tayber O. V. Errada P. R. Grant J. R.. (1999). Human CARD4 Protein Is a Novel CED-4/Apaf-1 Cell Death Family Member That Activates NF-κb. J. Biol. Chem. 274, 12955–12958. doi: 10.1074/jbc.274.19.12955 10224040

[B10] Bonhomme D. Santecchia I. Vernel-Pauillac F. Caroff M. Germon P. Murray G.. (2020). Leptospiral LPS Escapes Mouse TLR4 Internalization and TRIF−associated Antimicrobial Responses Through O Antigen and Associated Lipoproteins. PloS Pathogens. 16, e1008639. doi: 10.1371/journal.ppat.1008639 33362240 PMC7757799

[B11] Bonhomme D. Werts C. (2020). “Purification of LPS From Leptospira,” in Leptospira Spp, vol. pp . Eds. Koizumi N. Picardeau M. (New York, NY: Springer US), 53–65. doi: 10.1007/978-1-0716-0459-5_6 32632859

[B12] Botos I. Segal D. M. Davies D. R. (2011). The Structural Biology of Toll-Like Receptors. Structure. 19, 447–459. doi: 10.1016/j.str.2011.02.004 21481769 PMC3075535

[B13] Brough D. Le Feuvre R. A. Wheeler R. D. Solovyova N. Hilfiker S. Rothwell N. J.. (2003). Ca ^2+^ Stores and Ca ^2+^ Entry Differentially Contribute to the Release of IL-1β and IL-1α From Murine Macrophages. J. Immunol. 170, 3029–3036. doi: 10.4049/jimmunol.170.6.3029 12626557

[B14] Cameron C. E. (2015). “Leptospiral Structure, Physiology, and Metabolism,” in Leptospira and Leptospirosis, vol. pp . Ed. Adler B. (Berlin, Heidelberg: Springer Berlin Heidelberg), 21–41. doi: 10.1007/978-3-662-45059-8_3

[B15] Carneiro L. A. M. Travassos L. H. Girardin S. E. (2007). Nod-Like Receptors in Innate Immunity and Inflammatory Diseases. Ann. Med. 39, 581–593. doi: 10.1080/07853890701576172 18038361

[B16] Charon N. W. Lawrence C. W. O’Brien S. (1981). Movement of Antibody-Coated Latex Beads Attached to the Spirochete Leptospira Interrogans. Proc. Natl. Acad. Sci. U.S.A. 78, 7166–7170. doi: 10.1073/pnas.78.11.7166 6947280 PMC349217

[B17] Chassin C. Picardeau M. Goujon J.-M. Bourhy P. Quellard N. Darche S.. (2009). TLR4- and TLR2-Mediated B Cell Responses Control the Clearance of the Bacterial Pathogen, Leptospira Interrogans. J. Immunol. 183, 2669–2677. doi: 10.4049/jimmunol.0900506 19635914

[B18] Chen C. Zibiao H. Ming Z. Shiyi C. Ruixia L. Jie W.. (2014). Expression Pattern of Toll-Like Receptors (TLRs) in Different Organs and Effects of Lipopolysaccharide on the Expression of TLR 2 and 4 in Reproductive Organs of Female Rabbit. Dev. Comp. Immunol. 46, 341–348. doi: 10.1016/j.dci.2014.05.008 24858029

[B19] Cinco M. Banfi E. Panfili E. (1986). Heterogeneity of Lipopolysaccharide Banding Patterns in Leptospira Spp. Microbiol. 132, 1135–1138. doi: 10.1099/00221287-132-4-1135 3760822

[B20] Costa F. Hagan J. E. Calcagno J. Kane M. Torgerson P. Martinez-Silveira M. S.. (2015). Global Morbidity and Mortality of Leptospirosis: A Systematic Review. PloS Negl. Trop. Diseases. 9, e0003898. doi: 10.1371/journal.pntd.0003898 PMC4574773 26379143

[B21] Costa F. Zeppelini C. G. Ribeiro G. S. Santos N. Reis R. B. Martins R. D.. (2021). Household Rat Infestation in Urban Slum Populations: Development and Validation of a Predictive Score for Leptospirosis. PloS Negl. Trop. Dis. 15, e0009154. doi: 10.1371/journal.pntd.0009154 33657101 PMC7959339

[B22] Cullen P. A. Haake D. A. Bulach D. M. Zuerner R. L. Adler B. (2003). LipL21 is a Novel Surface-Exposed Lipoprotein of Pathogenic Leptospira Species. Infect Immun. 71, 2414–2421. doi: 10.1128/IAI.71.5.2414-2421.2003 12704111 PMC153295

[B23] Delude R. L. Savedra R. Jr. Zhao H. Thieringer R. Yamamoto S. Fenton M. J.. (1995). CD14 Enhances Cellular Responses to Endotoxin Without Imparting Ligand-Specific Recognition. Proc Natl Acad Sci U. S. A. 2 (20), 9288–9292. doi: 10.1073/pnas.92.20.9288 PMC40970 7568119

[B24] Desai S. van Treeck U. Lierz M. Espelage W. Zota L. Sarbu A.. (2009). Resurgence of Field Fever in a Temperate Country: An Epidemic of Leptospirosis Among Seasonal Strawberry Harvesters in Germany in 2007. Clin. Infect. Dis. 48, 691–697. doi: 10.1086/597036 19193108

[B25] Dick M. S. Sborgi L. Rühl S. Hiller S. Broz P. (2016). ASC Filament Formation Serves as a Signal Amplification Mechanism for Inflammasomes. Nat. Commun. 7, 11929. doi: 10.1038/ncomms11929 27329339 PMC4917984

[B26] Donnelly M. A. Steiner T. S. (2002). Two Nonadjacent Regions in Enteroaggregative Escherichia Coli Flagellin Are Required for Activation of Toll-Like Receptor 5. J. Biol. Chem. 277, 40456–40461. doi: 10.1074/jbc.M206851200 12185085

[B27] Dyevoich A. M. Disher N. S. Haro M. A. Haas K. M. (2020). A TLR4–TRIF-Dependent Signaling Pathway is Required for Protective Natural Tumor-Reactive IgM Production by B1 Cells. Cancer Immunol. Immunother. 69, 2113–2124. doi: 10.1007/s00262-020-02607-7 32448982 PMC7529868

[B28] Ellis W. Bryson D. Neill S. McParland P. Malone F. (1983). Possible Involvement of Leptospires in Abortion, Stillbirths and Neonatal Deaths in Sheep. Vet. Rec. 112, 291–293. doi: 10.1136/vr.112.13.291 6845608

[B29] Ellis W. Bryson D. O’Brien J. Neill S. (1983). Leptospiral Infection in Aborted Equine Foetuses. Equine Vet J. 15, 321–324. doi: 10.1111/j.2042-3306.1983.tb01811.x 6357776

[B30] Ellis W. McParland P. Bryson D. Cassells J. (1986). Prevalence of Leptospira Infection in Aborted Pigs in Northern Ireland. Vet. Rec. 118, 63–65. doi: 10.1136/vr.118.3.63 3952941

[B31] Ellis W. O’Brien J. Bryson D. Mackie D. (1985). Bovine Leptospirosis: Some Clinical Features of Serovar Hardjo Infection. Vet. Rec. 117, 101–104. doi: 10.1136/vr.117.5.101 4049693

[B32] Ellis W. Songer J. Montgomery J. Cassells J. (1986). Prevalence of Leptospira Interrogans Serovar Hardjo in the Genital and Urinary Tracts of non-Pregnant Cattle. Vet. Rec. 118, 11–13. doi: 10.1136/vr.118.1.11 3511601

[B33] Erridge C. Bennett-Guerrero E. Poxton I. R. (2002). Structure and Function of Lipopolysaccharides. Microbes Infect. 4, 837–851. doi: 10.1016/S1286-4579(02)01604-0 12270731

[B34] Eshghi A. Henderson J. Trent M. S. Picardeau M. (2015). *Leptospira Interrogans lpxD* Homologue Is Required for Thermal Acclimatization and Virulence. Infect Immun. 83, 4314–4321. doi: 10.1128/IAI.00897-15 26283339 PMC4598399

[B35] Faber E. Tedin K. Speidel Y. Brinkmann M. M. Josenhans C. (2018). Functional Expression of TLR5 of Different Vertebrate Species and Diversification in Intestinal Pathogen Recognition. Sci. Rep. 8, 11287. doi: 10.1038/s41598-018-29371-0> PMC6062626 30050158

[B36] Fanton d’Andon M. Quellard N. Fernandez B. Ratet G. Lacroix-Lamandé S. Vandewalle A.. (2014). Leptospira Interrogans Induces Fibrosis in the Mouse Kidney Through Inos-Dependent, TLR- and NLR-Independent Signaling Pathways. PloS Negl. Trop. Diseases. 8, e2664. doi: 10.1371/journal.pntd.0002664 PMC3907306 24498450

[B37] Ferrer M. F. Scharrig E. Charo N. Rípodas A. L. Drut R. Carrera Silva E. A.. (2018). Macrophages and Galectin 3 Control Bacterial Burden in Acute and Subacute Murine Leptospirosis That Determines Chronic Kidney Fibrosis. Front. Cell Infect. Microbiol. 8. doi: 10.3389/fcimb.2018.00384 PMC6218566 30425972

[B38] Forstnerič V. Ivičak-Kocjan K. Ljubetič A. Jerala R. Benčina M. (2016). Distinctive Recognition of Flagellin by Human and Mouse Toll-Like Receptor 5. PloS One 11, e0158894. doi: 10.1371/journal.pone.0158894 27391968 PMC4938411

[B39] Franchi L. Eigenbrod T. Núñez G. (2009). Cutting Edge: TNF-α Mediates Sensitization to ATP and Silica *via* the NLRP3 Inflammasome in the Absence of Microbial Stimulation. J. Immunol. 183, 792–796. doi: 10.4049/jimmunol.0900173 19542372 PMC2754237

[B40] Frey E. A. (1992). Soluble CD14 Participates in the Response of Cells to Lipopolysaccharide. J. Exp. Med. 176, 1665–1671. doi: 10.1084/jem.176.6.1665 1281215 PMC2119444

[B41] Fritz J. H. Ferrero R. L. Philpott D. J. Girardin S. E. (2006). Nod-Like Proteins in Immunity, Inflammation and Disease. Nat. Immunol. 7, 1250–1257. doi: 10.1038/ni1412 17110941

[B42] Gibson K. H. Trajtenberg F. Wunder E. A. Brady M. R. San Martin F. Mechaly A.. (2020). An Asymmetric Sheath Controls Flagellar Supercoiling and Motility in the Leptospira Spirochete. eLife. 9, e53672. doi: 10.7554/eLife.53672 32157997 PMC7065911

[B43] Girardin S. E. Boneca I. G. Viala J. Chamaillard M. Labigne A. Thomas G.. (2003). Nod2 Is a General Sensor of Peptidoglycan Through Muramyl Dipeptide (MDP) Detection. J. Biol. Chem. 278, 8869–8872. doi: 10.1074/jbc.C200651200 12527755

[B44] Girardin S. E. Jéhanno M. Mengin-Lecreulx D. Sansonetti P. J. Alzari P. M. Philpott D. J. (2005). Identification of the Critical Residues Involved in Peptidoglycan Detection by Nod1. J. Biol. Chem. 280, 38648–38656. doi: 10.1074/jbc.M509537200 16172124

[B45] Gomes C. K. Guedes M. Potula H.-H. Dellagostin O. A. Gomes-Solecki M. (2018). Sex Matters: Male Hamsters Are More Susceptible to Lethal Infection With Lower Doses of Pathogenic Leptospira Than Female Hamsters. Infect. Immun. 86, e00369–e00318. doi: 10.1128/IAI.00369-18 30012637 PMC6204738

[B46] Guo Y. Ding C. Zhang B. Xu J. Xun M. Xu J. (2016). Inhibitory Effect of BMAP-28 on Leptospiral Lipopolysaccharide-Induced TLR2-Dependent Immune Response in Bovine Cells. Jundishapur J. Microbiol. 9. doi: 10.5812/jjm.33926 PMC5013549 27635213

[B47] Guo Y. Fukuda T. Nakamura S. Bai L. Xu J. Kuroda K.. (2015). Interaction Between Leptospiral Lipopolysaccharide and Toll-Like Receptor 2 in Pig Fibroblast Cell Line, and Inhibitory Effect of Antibody Against Leptospiral Lipopolysaccharide on Interaction. Asian-Australasian J. Anim. Sci. 28, 273. doi: 10.5713/ajas.14.0440 PMC4283174 25557825

[B48] Haake D. A. Chao G. Zuerner R. L. Barnett J. K. Barnett D. Mazel M.. (2000). The Leptospiral Major Outer Membrane Protein LipL32 is a Lipoprotein Expressed During Mammalian Infection. Infect immun. 68, 2276–2285. doi: 10.1128/IAI.68.4.2276-2285.2000 10722630 PMC97414

[B49] Haake D. A. Zückert W. R. (2015). The Leptospiral Outer Membrane. Curr. Top. Microbiol. Immunol. 387, 187–221. doi: 10.1007/978-3-662-45059-8_8 25388136 PMC4419373

[B50] Hailman E. Lichenstein H. S. Wurfel M. M. Miller D. S. Johnson D. A. Kelley M.. (1994). Lipopolysaccharide (LPS)-Binding Protein Accelerates the Binding of LPS to CD14. J. Exp. Med. 179, 269–277. doi: 10.1084/jem.179.1.269 7505800 PMC2191344

[B51] Hamond C. Silveira C. S. Buroni F. Suanes A. Nieves C. Salaberry X.. (2019). *Leptospira Interrogans* Serogroup Pomona Serovar Kennewicki Infection in Two Sheep Flocks With Acute Leptospirosis in Uruguay. Transbound Emerg. Dis. 66, 1186–1194. doi: 10.1111/tbed.13133 30685885

[B52] Hathaway S. C. Blackmore D. K. (1981). Ecological Aspects of the Epidemiology of Infection With Leptospires of the Ballum Serogroup in the Black Rat ( *Rattus Rattus* ) and the Brown Rat ( *Rattus Norvegicus* ) in New Zealand. J. Hyg. 87, 427–436. doi: 10.1017/S0022172400069679 7310125 PMC2134120

[B53] Hayashi F. Smith K. D. Ozinsky A. Hawn T. R. Yi E. C. Goodlett D. R.. (2001). The Innate Immune Response to Bacterial Flagellin is Mediated by Toll-Like Receptor 5. Nature 410 (6832), 1099–1103. doi: 10.1038/35074106 11323673

[B54] He Y. Hara H. Núñez G. (2016). Mechanism and Regulation of NLRP3 Inflammasome Activation. Trends Biochem. Sci. 41, 1012–1021. doi: 10.1016/j.tibs.2016.09.002 27669650 PMC5123939

[B55] Heuser E. Fischer S. Ryll R. Mayer-Scholl A. Hoffmann D. Spahr C.. (2017). Survey for Zoonotic Pathogens in Norway Rat Populations From Europe: Survey for Zoonotic Pathogens in Norway Rat Populations From Europe. Pest Manag Sci. 73, 341–348. doi: 10.1002/ps.4339 27299665

[B56] Holzapfel M. Bonhomme D. Cagliero J. Vernel-Pauillac F. Fanton d’Andon M. Bortolussi S.. (2020). Escape of TLR5 Recognition by Leptospira Spp.: A Rationale for Atypical Endoflagella. Front. Immunol. 149, e229. doi: 10.3389/fimmu.2020.02007 PMC7431986 32849665

[B57] Holzapfel M. Taraveau F. Djelouadji Z. (2021). Serological and Molecular Detection of Pathogenic Leptospira in Domestic and Stray Cats on Reunion Island, French Indies. Epidemiol. Infect., 1–28. doi: 10.1017/S095026882100176X PMC8569831 34372952

[B58] Hsu S.-H. Chang M.-Y. Lin S.-M. Ko Y.-C. Chou L.-F. Tian Y.-C.. (2021). Peptidoglycan Mediates Leptospira Outer Membrane Protein Loa22 to Toll-Like Receptor 2 for Inflammatory Interaction: A Novel Innate Immune Recognition. Sci. Rep. 11, 1064. doi: 10.1038/s41598-020-79662-8 33441663 PMC8115183

[B59] Hsu S.-H. Hung C.-C. Chang M.-Y. Ko Y.-C. Yang H.-Y. Hsu H.-H.. (2017). Active Components of Leptospira Outer Membrane Protein LipL32 to Toll-Like Receptor 2. Sci. Rep. 7, 1–16. doi: 10.1038/s41598-017-08743-y 28827637 PMC5566480

[B60] Hsu S.-H. Lo Y.-Y. Tung J.-Y. Ko Y.-C. Sun Y.-J. Hung C.-C.. (2010). Leptospiral Outer Membrane Lipoprotein LipL32 Binding on Toll-Like Receptor 2 of Renal Cells As Determined With an Atomic Force Microscope. Biochemistry. 49, 5408–5417. doi: 10.1021/bi100058w 20513152

[B61] Jin M. S. Lee J.-O. (2008). Structures of the Toll-Like Receptor Family and Its Ligand Complexes. Immun. 29, 182–191. doi: 10.1016/j.immuni.2008.07.007 18701082

[B62] Kang J. Y. Nan X. Jin M. S. Youn S.-J. Ryu Y. H. Mah S.. (2009). Recognition of Lipopeptide Patterns by Toll-Like Receptor 2-Toll-Like Receptor 6 Heterodimer. Immun. 31, 873–884. doi: 10.1016/j.immuni.2009.09.018 19931471

[B63] Kawai T. Akira S. (2010). The Role of Pattern-Recognition Receptors in Innate Immunity: Update on Toll-Like Receptors. Nat. Immunol. 11 (5):373–384. doi: 10.1038/ni.1863 20404851

[B64] Keestra A. M. de Zoete M. R. van Aubel R. A. van Putten J. P. (2008). Functional Characterization of Chicken TLR5 Reveals Species-Specific Recognition of flagellin. Mol. Immunol. 45 (5), 1298–1307. doi: 10.1016/j.molimm.2007.09.013 17964652

[B65] Keestra A. M. de Zoete M. R. van Aubel R. A. M. H. van Putten J. P. M. (2007). The Central Leucine-Rich Repeat Region of Chicken TLR16 Dictates Unique Ligand Specificity and Species-Specific Interaction With TLR2. J. Immunol. 178 (11), 7110–7119. doi: 10.4049/jimmunol.178.11.7110 17513760

[B66] Kobayashi K. Inohara N. Hernandez L. D. Galán J. E. Núñez G. Janeway C. A.. (2002). RICK/Rip2/CARDIAK Mediates Signalling for Receptors of the Innate and Adaptive Immune Systems. Nat 416, 194–199. doi: 10.1038/416194a 11894098

[B67] Ko A. I. Goarant C. Picardeau M. (2009). Leptospira: The Dawn of the Molecular Genetics Era for an Emerging Zoonotic Pathogen. Nat. Rev. Microbiol. 7, 736–747. doi: 10.1038/nrmicro2208 19756012 PMC3384523

[B68] Lacroix-Lamande S. Fanton d’Andon M. Michel E. Ratet G. Philpott D. J. Girardin S. E.. (2012). Downregulation of the Na/K-ATPase Pump by Leptospiral Glycolipoprotein Activates the NLRP3 Inflammasome. J. Immunol. 188, 2805–2814. doi: 10.4049/jimmunol.1101987 22323544

[B69] Larsson C. Santa Rosa C. Larsson M. Birgel E. Fernandes W. Paim G. (1985). Laboratory and Clinical Features of Experimental Feline Leptospirosis. Int. J. Zoonoses. 12, 111–119. 4077410

[B70] Lawlor K. E. Vince J. E. (2014). Ambiguities in NLRP3 Inflammasome Regulation: Is There a Role for Mitochondria? Biochim. Biophys. Acta (BBA) - Gen. Subj. 1840, 1433–1440. doi: 10.1016/j.bbagen.2013.08.014 23994495

[B71] Leonvizcaino L. Demendoza M. Garrido F. (1987). Incidence of Abortions Caused by Leptospirosis in Sheep and Goats in Spain. Comp. Immunol Microbiol. Infect. Diseases. 10, 149–153. doi: 10.1016/0147-9571(87)90009-9 3304822

[B72] Lilenbaum W. Varges R. Brandão F. Z. Cortez A. de Souza S. O. Brandão P. E.. (2008). Detection of Leptospira Spp. In Semen and Vaginal Fluids of Goats and Sheep by Polymerase Chain Reaction. Theriogenology. 69, 837–842. doi: 10.1016/j.theriogenology.2007.10.027 18291518

[B73] Li S. Wang M. Ojcius D. M. Zhou B. Hu W. Liu Y.. (2018). Leptospira Interrogans Infection Leads to IL-1β and IL-18 Secretion From a Human Macrophage Cell Line Through Reactive Oxygen Species and Cathepsin B Mediated-NLRP3 Inflammasome Activation. Microbes Infect. 20, 254–260. doi: 10.1016/j.micinf.2018.01.010 29432801

[B74] Li S. Wang M. Ojcius D. M. Zhou B. Hu W. Liu Y.. (2021). Corrigendum to “Leptospira Interrogans Infection Leads to IL-1β and IL-18 Secretion From a Human Macrophage Cell Line Through Reactive Oxygen Species and Cathepsin B Mediated-NLRP3 Inflammasome Activation” [Microbe Infect, (2018) 254–260]. Microbes Infect. 23, 104756. doi: 10.1016/j.micinf.2020.09.002 32988716

[B75] Lo Y.-Y. Hsu S.-H. Ko Y.-C. Hung C.-C. Chang M.-Y. Hsu H.-H.. (2013). Essential Calcium-Binding Cluster of Leptospira LipL32 Protein for Inflammatory Responses Through the Toll-Like Receptor 2 Pathway. J. Biol. Chem. 288, 12335–12344. doi: 10.1074/jbc.M112.418699 23486465 PMC3636917

[B76] Lourdault K. Aviat F. Picardeau M. (2009). Use of Quantitative Real-Time PCR for Studying the Dissemination of Leptospira Interrogans in the Guinea Pig Infection Model of Leptospirosis. J. Med. Microbiol. 58, 648–655. doi: 10.1099/jmm.0.008169-0 19369528

[B77] Lozano-Aponte J. Scior T. Ambrosio F. N. M. González-Melchor M. Alexander C. (2020). Exploring Electrostatic Patterns of Human, Murine, Equine and Canine TLR4/MD-2 Receptors. Innate Immun. 26, 364–380. doi: 10.1177/1753425919894628 31874581 PMC7903528

[B78] Magalhaes J. G. Philpott D. J. Nahori M. Jéhanno M. Fritz J. Bourhis L.. (2005). Murine Nod1 But Not its Human Orthologue Mediates Innate Immune Detection of Tracheal Cytotoxin. EMBO Rep. 6, 1201–1207. doi: 10.1038/sj.embor.7400552 16211083 PMC1369207

[B79] Malkiel S. Kuhlow C. J. Mena P. Benach J. L. (2009). The Loss and Gain of Marginal Zone and Peritoneal B Cells Is Different in Response to Relapsing Fever and Lyme Disease Borrelia. J. Immunol. 182, 498–506. doi: 10.4049/jimmunol.182.1.498 19109181

[B80] Malmström J. Beck M. Schmidt A. Lange V. Deutsch E. W. Aebersold R. (2009). Proteome-Wide Cellular Protein Concentrations of the Human Pathogen Leptospira Interrogans. Nat 460, 762–765. doi: 10.1038/nature08184 PMC2723184 19606093

[B81] Matsui M. Roche L. Geroult S. Soupé-Gilbert M.-E. Monchy D. Huerre M.. (2016). Cytokine and Chemokine Expression in Kidneys During Chronic Leptospirosis in Reservoir and Susceptible Animal Models. PloS One 11, e0156084. doi: 10.1371/journal.pone.0156084 27219334 PMC4878748

[B82] Medzhitov R. Preston-Hurlburt P. Janeway C. A. (1997). A Human Homologue of the Drosophila Toll Protein Signals Activation of Adaptive Immunity. Nat 388, 394–397. doi: 10.1038/41131 9237759

[B83] Meng J. Drolet J. R. Monks B. G. Golenbock D. T. (2010). MD-2 Residues Tyrosine 42, Arginine 69, Aspartic Acid 122, and Leucine 125 Provide species-specificity for Lipid IVA. J. Biol. Chem. 285 (36), 27935–27943. doi: 10.1074/jbc.M110.134668 20592019 PMC2934660

[B84] Metcalfe H. J. La Ragione R. M. Smith D. G. E. Werling D. (2014). Functional Characterisation of Bovine TLR5 Indicates Species-Specific Recognition of Flagellin. Vet Immunol. Immunopathol. 157, 197–205. doi: 10.1016/j.vetimm.2013.12.006 24461722 PMC3969226

[B85] Miller D. K. Ayala J. M. Egger L. A. Raju S. M. Yamin T. T. Ding G. J.. (1993). Purification and Characterization of Active Human Interleukin-1 Beta-Converting Enzyme From THP.1 Monocytic Cells. J. Biol. Chem. 268, 18062–18069. doi: 10.1016/S0021-9258(17)46811-6 8349684

[B86] Moinet M. Wilkinson D. A. Aberdein D. Russell J. C. Vallée E. Collins-Emerson J. M.. (2021). Of Mice, Cattle, and Men: A Review of the Eco-Epidemiology of Leptospira Borgpetersenii Serovar Ballum. TropicalMed. 6, 189. doi: 10.3390/tropicalmed6040189 PMC8544700 34698305

[B87] Monte L. G. Jorge S. Xavier M. A. Leal F. M. A. Amaral M. G. Seixas F. K.. (2013). Molecular Characterization of Virulent Leptospira Interrogans Serogroup Icterohaemorrhagiae Isolated From Cavia Aperea. Acta Tropica. 126, 164–166. doi: 10.1016/j.actatropica.2013.02.009 23435256

[B88] Moore G. E. Guptill L. F. Glickman N. W. Caldanaro R. J. Aucoin D. Glickman L. T. (2006). Canine Leptospirosis, United States 2002–2004. Emerg. Infect. Dis. 12, 501–503. doi: 10.3201/eid1203.050809 16704794 PMC3291439

[B89] Muñoz-Planillo R. Kuffa P. Martínez-Colón G. Smith B. L. Rajendiran T. M. Núñez G. (2013). K+ Efflux Is the Common Trigger of NLRP3 Inflammasome Activation by Bacterial Toxins and Particulate Matter. Immun. 38, 1142–1153. doi: 10.1016/j.immuni.2013.05.016 PMC3730833 23809161

[B90] Nagel A. Vázquez C. L. Etulain J. Blanco F. C. Gravisaco M. J. Gómez R. M.. (2019). Bovine Macrophages Responses to the Infection With Virulent and Attenuated Leptospira Interrogans Serovar Pomona. Vet Microbiol. 233, 124–132. doi: 10.1016/j.vetmic.2019.04.033 31176398

[B91] Nahori M.-A. Fournié-Amazouz E. Que-Gewirth N. S. Balloy V. Chignard M. Raetz C. R. H.. (2005). Differential TLR Recognition of Leptospiral Lipid A and Lipopolysaccharide in Murine and Human Nells. J. Immunol. 175, 6022. doi: 10.4049/jimmunol.175.9.6022 16237097

[B92] Nair N. Guedes M. S. Hajjar A. M. Werts C. Gomes-Solecki M. (2021). Role of TLR4 in Persistent Leptospira Interrogans Infection: A Comparative *In Vivo* Study in Mice. Front. Immunol. 11. doi: 10.3389/fimmu.2020.572999 PMC7843520 33519799

[B93] Nally J. E. Fishbein M. C. Blanco D. R. Lovett M. A. (2005). Lethal Infection of C3H/HeJ and C3H/SCID Mice With an Isolate of *Leptospira Interrogans* Serovar Copenhageni. Infect. Immun. 73, 7014–7017. doi: 10.1128/IAI.73.10.7014-7017.2005 16177383 PMC1230959

[B94] Nalubamba K. S. Gossner A. G. Dalziel R. G. Hopkins J. (2007). Differential Expression of Pattern Recognition Receptors in Sheep Tissues and Leukocyte Subsets. Vet Immunol. Immunopathol. 118, 252–262. doi: 10.1016/j.vetimm.2007.05.018 17604125

[B95] Netea M. G. Nold-Petry C. A. Nold M. F. Joosten L. A. Opitz B. van de Meer J. H. (2009). Differential Requirement for the Activation of the Inflammasome for Processing and Release of IL-1␤ in Monocytes and Macrophages. Blood 113 (10), 2324–2335. doi: 10.1182/blood-2008-03-146720 19104081 PMC2652374

[B96] Novak A. Pupo E. van’t Veld E. Rutten V. P. M. G. Broere F. Sloots A. (2022). Activation of Canine, Mouse and Human TLR2 and TLR4 by Inactivated Leptospira Vaccine Strains. Front. Immunol. 13. doi: 10.3389/fimmu.2022.823058 PMC8978998 35386703

[B97] Osvaldova A. Woodman S. Patterson N. Offord V. Mwangi D. Gibson A. J.. (2014). Replacement of Two Aminoacids in the Bovine Toll-Like Receptor 5 TIR Domain With Their Human Counterparts Partially Restores Functional Response to Flagellin. Dev. Comp. Immunol. 47, 90–94. doi: 10.1016/j.dci.2014.07.002 25020193

[B98] Paiva-Cardoso M das N. Arent Z. Gilmore C. Hartskeerl R. Ellis W. A. (2013). Altodouro, a New Leptospira Serovar of the Pomona Serogroup Isolated From Rodents in Northern Portugal. Infect Genet. Evolution. 13, 211–217. doi: 10.1016/j.meegid.2012.09.013 23070280

[B99] Panda S. Ding J. L. (2015). Natural Antibodies Bridge Innate and Adaptive Immunity. JI. 194, 13–20. doi: 10.4049/jimmunol.1400844 25527792

[B100] Park B. S. Song D. H. Kim H. M. Choi B.-S. Lee H. Lee J.-O. (2009). The Structural Basis of Lipopolysaccharide Recognition by the TLR4–MD-2 Complex. Nat 458, 1191–1195. doi: 10.1038/nature07830 19252480

[B101] Patra K. P. Choudhury B. Matthias M. M. Baga S. Bandyopadhya K. Vinetz J. M. (2015). Comparative Analysis of Lipopolysaccharides of Pathogenic and Intermediately Pathogenic Leptospira Species. BMC Microbiol. 15, 244. doi: 10.1186/s12866-015-0581-7 26518696 PMC4628369

[B102] Pena-Moctezuma A. Bulach D. M. Kalambaheti T. Adler B. (1999). Comparative Analysis of the LPS Biosynthetic Loci of the Genetic Subtypes of Serovar Hardjo: Leptospira Interrogans Subtype Hardjoprajitno and Leptospira Borgpetersenii Subtype Hardjobovis. FEMS Microbiol. Lett. 177, 319–326. doi: 10.1111/j.1574-6968.1999.tb13749.x 10474199

[B103] Pereira M. M. Andrade J. Marchevsky R. S. Ribeiro dos Santos R. (1998). Morphological Characterization of Lung and Kidney Lesions Inc3h/HeJ Mice Infected With Leptospira Interrogans Serovar Icterohaemorrhagiae: Defect of CD4+ and CD8+ T-Cells are Prognosticators of the Disease Progression. Exp. Toxicol Pathol. 50, 191–198. doi: 10.1016/S0940-2993(98)80083-3 9681649

[B104] Poltorak A. (1998). Defective LPS Signaling in C3H/HeJ and C57BL/10ScCr Mice: Mutations in Tlr4 Gene. Sci. 282, 2085–2088. doi: 10.1126/science.282.5396.2085 9851930

[B105] Que-Gewirth N. L. S. Ribeiro A. A. Kalb S. R. Cotter R. J. Bulach D. M. Adler B.. (2004). A Methylated Phosphate Group and Four Amide-Linked Acyl Chains in Leptospira Interrogans Lipid A: The Membrane Anchor of an Unusual Lipopolysaccharide That Activates TLR2. J. Biol. Chem. 279, 25420–25429. doi: 10.1074/jbc.M400598200 15044492 PMC2556802

[B106] Raddi G. Morado D. R. Yan J. Haake D. A. Yang X. F. Liu J. (2012). Three-Dimensional Structures of Pathogenic and Saprophytic Leptospira Species Revealed by Cryo-Electron Tomography. J. Bacteriol. 194, 1299–1306. doi: 10.1128/JB.06474-11 22228733 PMC3294836

[B107] Rajeev S. Toka F. N. Shiokawa K. (2020). Potential Use of a Canine Whole Blood Culture System to Evaluate the Immune Response to Leptospira. Comp. Immunol Microbiol. Infect. Diseases. 73, 101546. doi: 10.1016/j.cimid.2020.101546 32916553

[B108] Ranoa D. R. E. Kelley S. L. Tapping R. I. (2013). Human Lipopolysaccharide-Binding Protein (LBP) and CD14 Independently Deliver Triacylated Lipoproteins to Toll-Like Receptor 1 (TLR1) and TLR2 and Enhance Formation of the Ternary Signaling Complex. J. Biol. Chem. 288, 9729. doi: 10.1074/jbc.M113.453266 23430250 PMC3617275

[B109] Ratet G. Santecchia I. Fanton d’Andon M. Vernel-Pauillac F. Wheeler R. Lenormand P.. (2017). LipL21 Lipoprotein Binding to Peptidoglycan Enables Leptospira Interrogans to Escape NOD1 and NOD2 Recognition. PloS Pathogens. 13, e1006725. doi: 10.1371/journal.ppat.1006725 29211798 PMC5764436

[B110] Ratet G. Veyrier F. J. Fanton d’Andon M. Kammerscheit X. Nicola M.-A. Picardeau M.. (2014). Live Imaging of Bioluminescent Leptospira Interrogans in Mice Reveals Renal Colonization as a Stealth Escape From the Blood Defenses and Antibiotics. PloS Negl. Trop. Diseases. 8, e3359. doi: 10.1371/journal.pntd.0003359 PMC4256284 25474719

[B111] Ren S.-X. Fu G. Jiang X.-G. Zeng R. Miao Y.-G. Xu H.. (2003). Unique Physiological and Pathogenic Features of Leptospira Interrogans Revealed by Whole-Genome Sequencing. Nat 422, 888–893. doi: 10.1038/nature01597 12712204

[B112] Rietschel E. T. Kirikae T. Schade F. U. Mamat U. Schmidt G. Loppnow H.. (1994). Bacterial Endotoxin: Molecular Relationships of Structure to Activity and Function. FASEB J. 8, 217–225. doi: 10.1096/fasebj.8.2.8119492 8119492

[B113] Rigby C. (1976). Natural Infections of Guinea-Pigs. Lab. Anim. 10, 119–142. doi: 10.1258/002367776781071503 180326

[B114] Ristow P. Bourhy P. McBride FW da C. Figueira C. P. Huerre M. Ave P.. (2007). The OmpA-Like Protein Loa22 is Rssential for Leptospiral Virulence. PloS Pathogens. 3, e97. doi: 10.1371/journal.ppat.0030097 17630832 PMC1914066

[B115] Ritter J. M. Lau C. Craig S. B. Goarant C. Nilles E. J. Ko A. I.. (2018). A Large Leptospirosis Outbreak Following Successive Severe Floods in Fij. Am. J. Trop. Med. Hygiene. 99, 849–851. doi: 10.4269/ajtmh.18-0335 PMC6159581 30141390

[B116] Ryu J.-K. Kim S. J. Rah S.-H. Kang J. I. Jung H. E. Lee D.. (2017). Reconstruction of LPS Transfer Cascade Reveals Structural Determinants Within LBP, CD14, and TLR4-MD2 for Efficient LPS Recognition and Transfer. Immun. 46, 38–50. doi: 10.1016/j.immuni.2016.11.007 27986454

[B117] Sansonetti P. J. Phalipon A. Arondel J. Thirumalai K. Banerjee S. Akira S.. (2000). Caspase-1 Activation of IL-1␤ and IL-18 Are Essential for Shigella Flexneri–Induced Inflammation. Immunity 12 (5), 581–590. doi: 10.1016/S1074-7613(00)80209-5 10843390

[B118] Sebek Z. Grulich I. Valova M. (1987). To the Knowledge of the Common Hamster (Cricetus Cricetus Linné, 1758; Rodentia) as a Host of Leptospirosis in Czechoslovajia. Folia Parasitol. (Praha). 34, 97–105. 3596398

[B119] Sellati T. J. Bouis D. A. Caimano M. J. Feulner J. A. Ayers C. Lien E.. (1999). Activation of Human Monocytic Cells by Borrelia Burgdorferi and Treponema Pallidum is Facilitated by CD14 and Correlates With Surface Exposure of Spirochetal Lipoproteins. J. Immunol. 163, 2049–2056. 10438943

[B120] Sellati T. J. Bouis D. A. Kitchens R. L. Darveau R. P. Pugin J. Ulevitch R. J.. (1998). Treponema Pallidum and Borrelia Burgdorferi Lipoproteins and Synthetic Lipopeptides Activate Monocytic Cells *via* a CD14-Dependent Pathway Distinct From That Used by Lipopolysaccharide. J. Immunol. 160, 5455–5464. 9605148

[B121] Senavirathna I. Rathish D. Agampodi S. (2020). Cytokine Response in Human Leptospirosis With Different Clinical Outcomes: A Systematic Review. BMC Infect. Dis. 20, 268. doi: 10.1186/s12879-020-04986-9 32264832 PMC7137275

[B122] Slamti L. de Pedro M. A. Guichet E. Picardeau M. (2011). Deciphering Morphological Determinants of the Helix-Shaped Leptospira. J. Bacteriol. 193, 6266–6275. doi: 10.1128/JB.05695-11 21926230 PMC3209227

[B123] Smith K. D. Andersen-Nissen E. Hayashi F. Strobe K. Bergman M. A. Barrett S. L. R.. (2003). Toll-Like Receptor 5 Recognizes a Conserved Site on Flagellin Required for Protofilament Formation and Bacterial Motility. Nat. Immunol. 4, 1247–1253. doi: 10.1038/ni1011 14625549

[B124] Subharat S. Wilson P. Heuer C. Collins-Emerson J. (2010). Investigation of Localisation of Leptospira Spp. In Uterine and Fetal Tissues of non-Pregnant and Pregnant Farmed Deer. null. 58, 281–285. doi: 10.1080/00480169.2010.69755 21151213

[B125] Su Q. Chen Y. Wang B. Zhang Q. He H. (2021). Genetic Characterization of Toll-Like Receptors in the Brown Rat and Their Association With Pathogen Infections. Integr. Zool. 0, 1–11. doi: 10.1111/1749-4877.12555 34003606

[B126] Tahoun A. Jensen K. Corripio-Miyar Y. McAteer S. Smith D. G. E. McNeilly T. N.. (2017). Host Species Adaptation of TLR5 Signalling and Flagellin Recognition. Sci. Rep. 7, 17677. doi: 10.1038/s41598-017-17935-5 29247203 PMC5732158

[B127] Teghanemt A. Zhang D. Levis E. N. Weiss J. P. Gioannini T. L. (2005). Molecular Basis of Reduced Potency of Underacylated Endotoxins. J. Immunol. 175, 4669–4676. doi: 10.4049/jimmunol.175.7.4669 16177114

[B128] Vincent A. T. Schiettekatte O. Goarant C. Neela V. K. Bernet E. Thibeaux R.. (2019). Revisiting the Taxonomy and Evolution of Pathogenicity of the Genus Leptospira Through the Prism of Genomics. PloS Negl. Trop. Dis. 13, e0007270. doi: 10.1371/journal.pntd.0007270 31120895 PMC6532842

[B129] Vinh T. Adler B. Faine S. (1986). Ultrastructure and Chemical Composition of Lipopolysaccharide Extracted From Leptospira Interrogans Serovar Copenhageni. J. Gen. Microbiol. 132, 103–109. doi: 10.1099/00221287-132-1-103 3711857

[B130] Viriyakosol S. Matthias M. A. Swancutt M. A. Kirkland T. N. Vinetz J. M. (2006). Toll-Like Receptor 4 Protects Against Lethal Leptospira Interrogans Serovar Icterohaemorrhagiae Infection and Contributes to *In Vivo* Control of Leptospiral Burden. IAI. 74, 887–895. doi: 10.1128/IAI.74.2.887-895.2006 PMC1360355 16428731

[B131] Vollmer W. Blanot D. De Pedro M. A. (2008). Peptidoglycan Structure and Architecture. FEMS Microbiol. Rev. 32, 149–167. doi: 10.1111/j.1574-6976.2007.00094.x 18194336

[B132] Wang H. Mao L. Meng G. (2013). The NLRP3 Inflammasome Activation in Human or Mouse Cells, Sensitivity Causes Puzzle. Protein Cell. 4, 565–568. doi: 10.1007/s13238-013-3905-0 23794000 PMC4875544

[B133] Werling D. Jann O. C. Offord V. Glass E. J. Coffey T. J. (2009). Variation Matters: TLR Structure and Species-Specific Pathogen Recognition. Trends Immunol. 30, 124–130. doi: 10.1016/j.it.2008.12.001 19211304

[B134] Werts C. Tapping R. I. Mathison J. C. Chuang T.-H. Kravchenko V. Saint Girons I.. (2001). Leptospiral Lipopolysaccharide Activates Cells Through a TLR2-Dependent Mechanism. Nat. Immunol. 2, 346–352. doi: 10.1038/86354 11276206

[B135] Wooten R. M. Morrison T. B. Weis J. H. Wright S. D. Thieringer R. Weis J. J. (1998). The Role of CD14 in Signaling Mediated by Outer Membrane Lipoproteins of Borrelia Burgdorferi. J. Immunol. 160, 5485–5492. 9605151

[B136] Wright S. Ramos R. Tobias P. Ulevitch R. Mathison J. (1990). CD14, a Receptor for Complexes of Lipopolysaccharide (LPS) and LPS Binding Protein. Sci. 249, 1431–1433. doi: 10.1126/science.1698311 1698311

[B137] Xia B. Sun L. Fan X. Xiao H. Zhu Y. Qin J.. (2017). A New Model of Self-Resolving Leptospirosis in Mice Infected With a Strain of Leptospira Interrogans Serovar Autumnalis Harboring LPS Signaling Only Through TLR4. Emerg. Microbes Infect. 6, e36. doi: 10.1038/emi.2017.16 28536433 PMC5520481

[B138] Yamin T.-T. Ayala J. M. Miller D. K. (1996). Activation of the Native 45-kDa Precursor Form of Interleukin-1-Converting Enzyme. J. Biol. Chem. 271, 13273–13282. doi: 10.1074/jbc.271.22.13273 8662843

[B139] Yang C.-W. Hung C.-C. Wu M.-S. Tian Y.-C. Chang C.-T. Pan M.-J.. (2006). Toll-Like Receptor 2 Mediates Early Inflammation by Leptospiral Outer Membrane Proteins in Proximal Tubule Cells. Kidney Int. 69, 815–822. doi: 10.1038/sj.ki.5000119 16437059

[B140] Yoon S.-I. Kurnasov O. Natarajan V. Hong M. Gudkov A. V. Osterman A. L.. (2012). Structural Basis of TLR5-Flagellin Recognition and Signaling. Sci. 335, 859–864. doi: 10.1126/science.1215584 PMC3406927 22344444

[B141] Zhang W. Xie X. Wu D. Jin X. Liu R. Hu X.. (2017). Doxycycline Attenuates Leptospira-Induced IL-1β by Suppressing NLRP3 Inflammasome Priming. Front. Immunol. 8. doi: 10.3389/fimmu.2017.00857 PMC5522854 28791016

[B142] Zhu A. Wei L. Hu S. Yang C. Chen C. Zhou Z.. (2020). Characterisation and Functional Analysis of Canine TLR5. Innate Immun. 26, 451–458. doi: 10.1177/1753425920901862 31986950 PMC7491235

